# *Candida albicans* triggers NADPH oxidase-independent neutrophil extracellular traps through dectin-2

**DOI:** 10.1371/journal.ppat.1008096

**Published:** 2019-11-06

**Authors:** Sheng-Yang Wu, Chia-Lin Weng, Min-Jhen Jheng, Hung-Wei Kan, Sung-Tsang Hsieh, Fu-Tong Liu, Betty A. Wu-Hsieh

**Affiliations:** 1 Graduate Institute of Immunology, National Taiwan University College of Medicine, Taipei, Taiwan; 2 Department of Anatomy and Cell Biology, National Taiwan University College of Medicine, Taipei, Taiwan; 3 Institute of Biomedical Science, Academia Sinica, Taipei, Taiwan; Memorial Sloan-Kettering Cancer Center, UNITED STATES

## Abstract

*Candida albicans* is one of the top leading causes of healthcare-associated bloodstream infection. Neutrophil extracellular traps (NET) are known to capture and kill pathogens. It is reported that opsonized *C*. *albicans*-triggered NETosis is NADPH oxidase-dependent. We discovered a NADPH oxidase-independent NETosis pathway in neutrophil response to unopsonized *C*. *albicans*. While CR3 engagement with opsonized *C*. *albicans* triggered NET, dectin-2 recognized unopsonized *C*. *albicans* and mediated NET formation. Engagement of dectin-2 activated the downstream Syk-Ca^2+^-PKCδ-protein arginine deiminase 4 (PAD4) signaling pathway which modulated nuclear translocation of neutrophil elastase (NE), histone citrullination and NETosis. In a *C*. *albicans* peritonitis model we observed Ki67^+^Ly6G^+^ NETotic cells in the peritoneal exudate and mesenteric tissues within 3 h of infection. Treatment with PAD4 inhibitor GSK484 or dectin-2 deficiency reduced % Ki67^+^Ly6G^+^ cells and the intensity of Ki67 in peritoneal neutrophils. Employing DNA digestion enzyme micrococcal nuclease, GSK484 as well as dectin-2-deficient mice, we further showed that dectin-2-mediated PAD4-dependent NET formation in vivo restrained the spread of *C*. *albicans* from the peritoneal cavity to kidney. Taken together, this study reveals that unopsonized *C*. *albicans* evokes NADPH oxidase-independent NETosis through dectin-2 and its downstream signaling pathway and dectin-2-mediated NET helps restrain fungal dissemination.

## Author summary

*Candida albicans* as a dimorphic fungal pathogen is one of the top leading causes of overall healthcare-associated bloodstream infection worldwide. Invasive candidiasis affects more than 250,000 people each year and leads to more than 50,000 deaths. Upon stimulation, neutrophils release nuclear DNA that forms a web-like structure named neutrophil extracellular traps (NET). NET is known to capture pathogens and restrain the spread of infection in the host. It has been reported that opsonized *C*. *albicans* induces NET through NADPH oxidase. Here we show a NADPH oxidase-independent NETosis in response to unopsonized *C*. *albicans*. Signaling pathway leading to NETosis involves dectin-2 downstream Syk-Ca^2+^-PKCδ-PAD4/NE. In a *C*. *albicans* peritonitis model, NETotic cells are found in the peritoneal exudates and they adhere to mesenteric tissue. Treatment with PAD4 inhibitor or dectin-2 deficiency dampens the ability of neutrophil to undergo NETosis and facilitates the spread of fungus from the peritoneal cavity to kidney. Our work defines the molecular mechanism involved in NADPH oxidase-independent NET formation and sheds light on the role of dectin-2 in neutrophil anti-*C*. *albicans* function.

## Introduction

*Candida albicans* is a commensal in the mucosa surface and skin in most humans. Environmental changes in temperature, nutrition, or the presence of serum induce its transformation from yeast to hyphae. *C*. *albicans* infection is one of the top leading causes of overall healthcare-associated bloodstream infection in medical centers as well as regional hospitals. Invasive candidiasis affects more than 250,000 people worldwide each year and leads to more than 50,000 deaths. Mortality among patients with invasive candidiasis is as high as 40% even after receiving antifungal therapy [1–3]. Patients with neutropenia or genetic deficiency in NADPH oxidase are susceptible to invasive candidiasis [4, 5], showing that neutrophils and NADPH oxidase activation are indispensable for host defense against *C*. *albicans* infection.

NADPH oxidase activation requires the assembly of its regulatory subunits, p40^phox^, p47^phox^, and p67^phox^ with its core proteins gp91^phox^ and p22^phox^, resulting in ROS production [6, 7]. In addition to generating ROS, NADPH oxidase activation also induces neutrophil release of nuclear DNA to form a sticky web-like structure named neutrophil extracellular trap (NET) that binds histones, granular proteins and antimicrobial peptides [8]. In vitro studies showed that pathogens trapped by NET are in contact with and killed by concentrated antimicrobial factors [9, 10]. NET is known to capture and kill *C*. *albicans* through a NADPH oxidase-dependent mechanism [11]. However, it is also reported that human neutrophils are capable of killing unopsonized *C*. *albicans* through a ROS-independent mechanism [12]. Whether *C*. *albicans* can induce NET through a NADPH oxidase-independent mechanism is a question to be addressed.

The process of NET formation is called NETosis. Neutrophils undergoing NETosis are characterized by disintegrated nuclear envelop and release of decondensed chromatin into the cytoplasm [8]. Recent study uncovers that cell cycle pathway controls NETosis. NETotic neutrophils have phosphorylated retinoblastoma protein and lamins and express cell cycle marker Ki67 [13]. Chromatin decondensation is the result of protein arginine deiminase 4 (PAD4)-dependent histone citrullination and neutrophil elastase (NE)-mediated histone degradation [14, 15]. NADPH oxidase facilitates both nuclear translocation of NE and PAD4 activation through stimulating myeloperoxidase activation [16]. It has been shown that opsonized *C*. *albicans* induces NET through autophagy, ROS, and NE, but not PAD4, apoptosis nor necroptosis [17]. Unopsonized *C*. *albicans* is also known to induce NET formation [11]. Since neutrophils use different receptors to recognize serum-opsonized and unopsonized *C*. *albicans* [12], it is important to investigate the receptor and the molecular mechanism by which unopsonized *C*. *albicans* uses to evoke NET.

Multiple receptors participate in modulating neutrophil anti-*C*. *albicans* functions. Fcγ receptor mediates human neutrophil killing of antibody-opsonized fungus through the Syk and PKC signaling pathways, whereas complement receptor 3 (CR3) are involved in killing of unopsonized *C*. *albicans* [12]. Mouse neutrophils utilize CR3 for recognition and killing of opsonized *C*. *albicans* [18]. Dectin-2 is marginally involved in opsonized *C*. *albicans*-induced neutrophil ROS production [19]. Dectin-1 as a phagocytosis receptor for *C*. *albicans* yeasts negatively regulates NETosis through interfering with nuclear translocation of granule NE [20]. Although CR3 as a receptor recognizing fibronectin-coated matrix is responsible for *C*. *albicans*-induced NETosis [21], which receptor(s) mediates NET formation in response to unopsonized *C*. *albicans* alone awaits to be determined.

Here we sought to study the receptor and signaling pathway that mediate unopsonized *C*. *albicans*-induced NET formation. Our study revealed the role of dectin-2 and its downstream Syk-Ca^2+^-PKCδ-PAD4/NE pathway in inducing NETosis in a NADPH oxidase-independent manner. Dectin-2 functions to restrain *C*. *albicans* spread from peritoneal cavity to kidney through modulating NET.

## Results

### Both opsonized and unopsonized *C*. *albicans* induce NET formation

**Fig 1:**
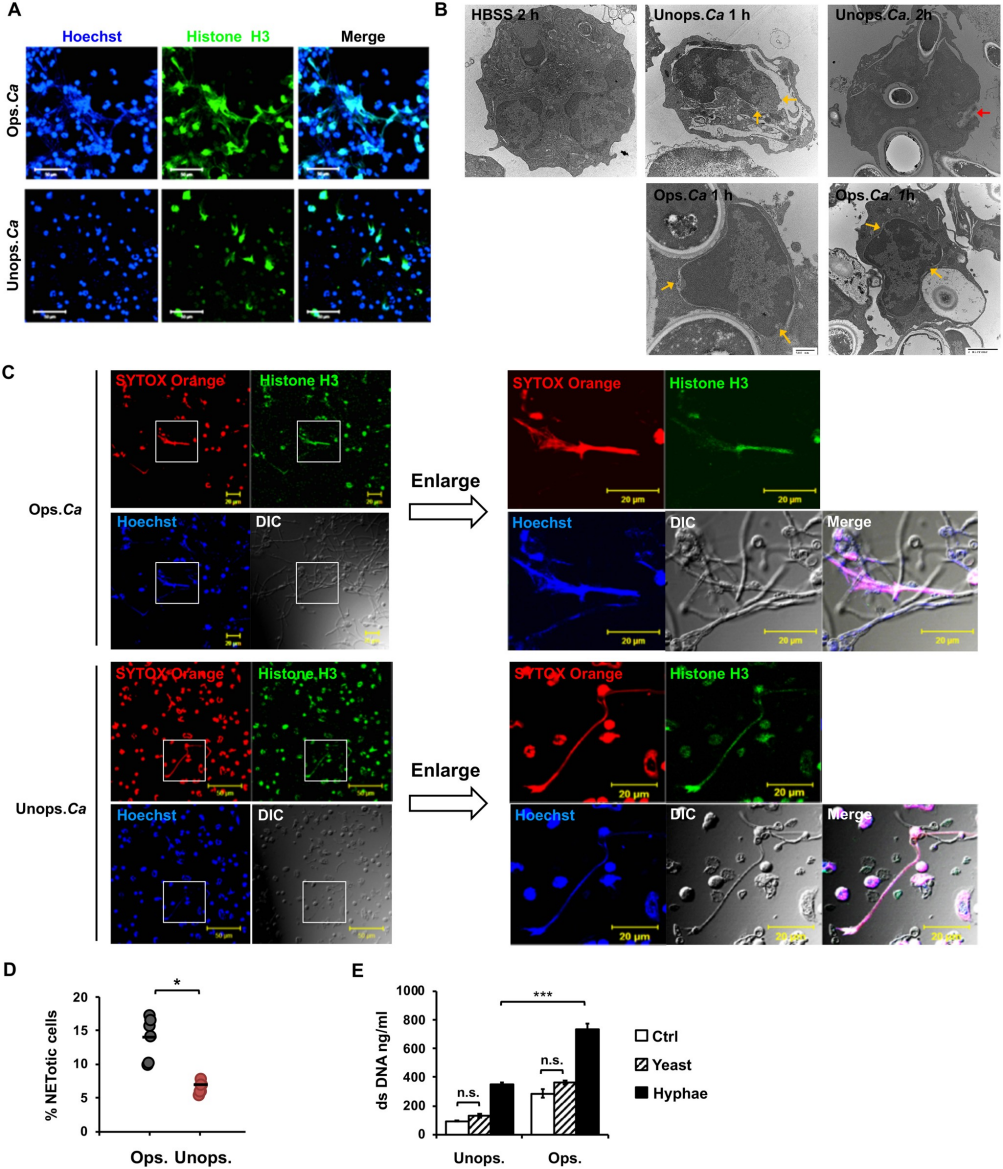
Both opsonized and unopsonized *C*. *albicans* induce NETosis. Neutrophils were stimulated or not (Ctrl) with opsonized (Ops.) and unopsonized (Unops.) *C*. *albicans* (*Ca*) at MOI of 2. (A) Cells were stained with anti-histone H3 antibody and Hoechst 33342. Immunofluorescence images were viewed under confocal microscope at 3 h of stimulation. (B) Transmission electron microscopy of unstimulated neutrophils (HBSS 0 h) or neutrophils stimulated with unopsonized *C*. *albicans* for 1 h (Unops.*Ca* 1 h) or 2 h (Unops.*Ca* 2 h) or with opsonized *C*. *albicans* for 1 h (Ops.*Ca* 1 h). Yellow arrows point to disintegrated nuclear envelop, whereas the red arrow points to disrupted cell membrane where cytosolic materials are being released. (C) Cells were stained with anti-histone H3 antibody (green), cell-impermeable DNA dye SYTOX Orange (red), and cell-permeable DNA dye Hoechst 33342 (blue). Immunofluorescence images were viewed under fluorescence microscope at 3 h of stimulation. DIC, differential interference contrast image. Images in the boxed areas are enlarged and shown on the right. (D) % NETotic cells = the number of cells that had NET morphology (SYTOX Orange^+^, web-like structure) after stimulation with opsonized (Ops) or unopsonized (Unops.) *C*. *albicans* divided by the total number of cells (blue) counted in images as prepared in (C). (E) *C*. *albicans* strains HLC 54 (yeast-locked) and SC 5314 (germination-competent) were incubated in RPMI medium for 4 h to allow competent cells to germinate. Neutrophils were then stimulated with opsonized and unopsonized HLC 54 (Yeast) and SC 5314 (Hyphae). Ctrl for the unops. group was cells incubated in HBSS only. Ctrl for the ops. group was cells incubated in HBSS containing 5% mouse serum. Extracellular DNA was quantified by Quant-iT PicoGreen dsDNA assay (n = 3). *, *p* < 0.05; ***, *p* < 0.005; n.s., not significant, as analyzed by Student’s *t* test.

It has been reported that opsonized *C*. *albicans* induces NETosis [17, 22, 23]. Our results revealed that neutrophils released web-like extracellular DNA fibers in response to opsonized as well as unopsonized *C*. *albicans* (Fig 1A). NETosis is characterized by disintegration of the nuclear envelope [8]. While stimulation by opsonized *C*. *albicans* resulted in nuclear membrane disintegration (Fig 1B), we also observed nuclear envelope breakdown and cytoplasmic membrane rupture following stimulation by unopsonized *C*. *albicans* (Fig 1B). Fluorescence images at high magnification clearly demonstrated that similar to opsonized *C*. *albicans* stimulation, unopsonized *C*. *albicans* hyphae were entangled with histone H3-containing web-like extracellular DNA structure (Fig 1C), although the percentage of NETotic cells in response to unopsonized *C*. *albicans* (6.8%) was lower than that to opsonized organisms (13.9%) (Fig 1D). Since opsonin-containing fresh serum also facilitates the germination of *C*. *albicans*, we allowed *C*. *albicans* yeasts to germinate before adding them to wells containing neutrophils. PicoGreen dsDNA assay showed that the hyphal form but not yeast-locked *C*. *albicans* (strain HLC 54) induced NETosis whether it was opsonized or not (Fig 1E), signifying the importance of hyphal formation in triggering NETosis. Our data indicate that not only opsonized but also unopsonized *C*. *albicans* in its hyphal form induces NETosis.

### Unopsonized *C*. *albicans*-induced NET formation is independent of NCF-1

**Fig 2:**
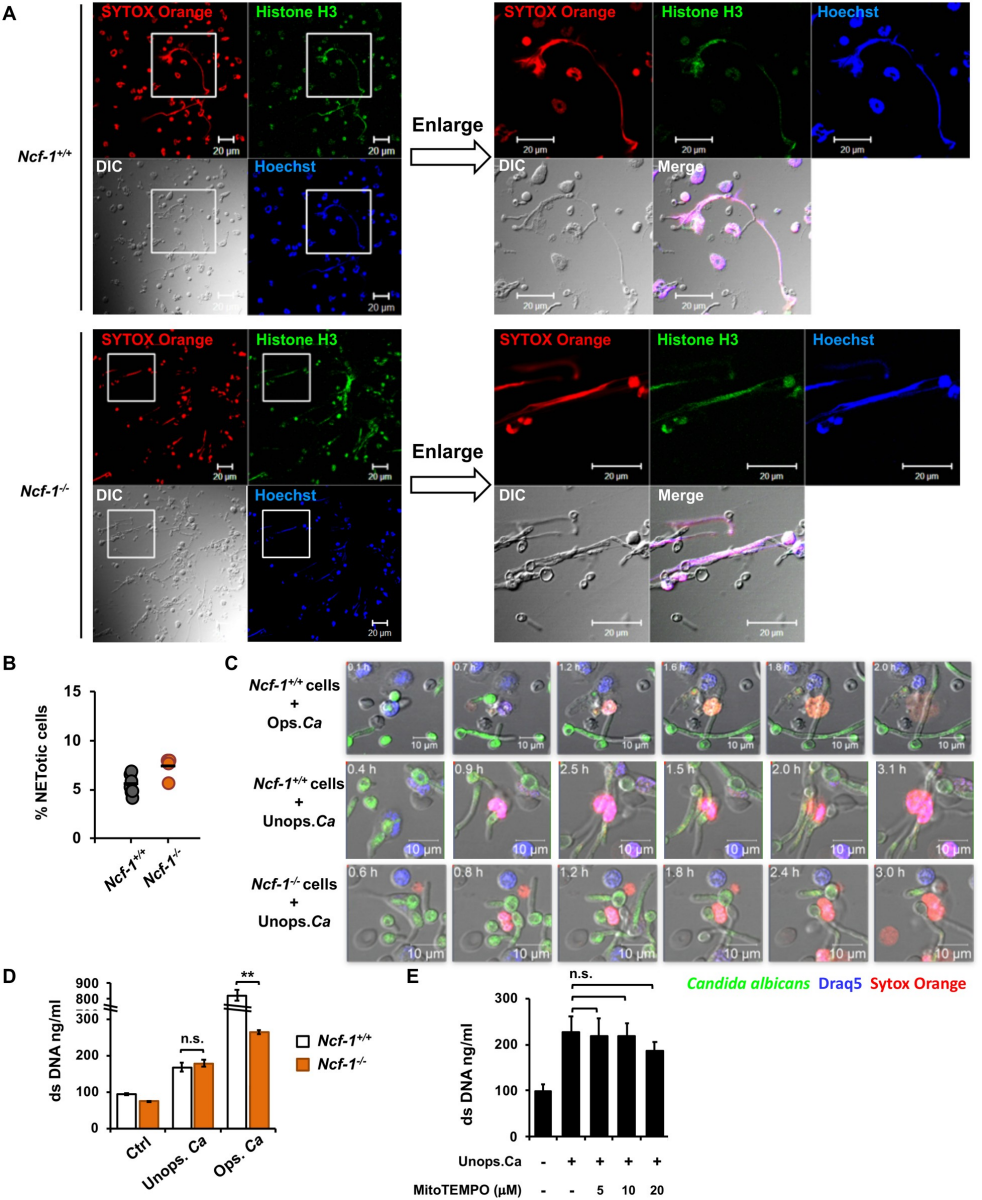
Unopsonized *C*. *albicans*-induced NET formation is independent of NCF-1- and mitochondrial ROS. *Ncf-1*^*+/+*^ and *Ncf-1*^*-/-*^ neutrophils were stimulated or not (Ctrl) with opsonized (Ops.) or unopsonized (Unops.) *C*. *albicans* at MOI of 2. (A) Cells were stimulated with unopsonized *C*. *albicans* for 3 h and stained with anti-histone H3 antibody (green), cell-impermeable DNA dye SYTOX Orange (red), and cell-permeable DNA dye Hoechst 33342 (blue). Immunofluorescence images were viewed under fluorescence microscope. DIC, differential interference contrast image. Images in the boxed areas are enlarged and shown on the right. (B) % NETotic cells = the number of cells that had NET morphology (SYTOX Orange^+^, web-like structure) divided by the total number of cells (blue) counted in images as prepared in (A). (C) Live cells were stained with cell-permeable DNA dye Draq5 (blue) and cell-impermeable DNA dye SYTOX Orange (red) before stimulation with GFP-expressing *C*. *albicans* strain OG1 (green). NETosis in response to opsonized and unopsonized pre-germinated *C*. *albicans* was observed over 180 min after addition of *C*. *albicans*. Zeiss LSM 780 confocal microscope was employed for time-lapse imaging (Images were obtained from S1, S2 and S3 Videos separately). (D) Cells were stimulated with opsonized and unopsonized *C*. *albicans* for 3 h. Cell supernatants were collected for Quant-iT PicoGreen dsDNA assay. (n = 3) (E) Cells were pretreated with 5, 10, 20 μM of mitochondria ROS inhibitor, MitoTEMPO, for 30 min before stimulation with unopsonized *C*. *albicans*. Cell supernatants were collected for Quant-iT PicoGreen dsDNA assay. (n = 3). All experiments were performed three times. Data from one representative experiment are presented as mean ± standard deviation (SD). **, *p <* 0.01; n.s., not significant, as analyzed by Student’s *t* test.

We further explored the requirement of ROS in unopsonized *C*. *albicans*-induced NET formation. Results showed that NCF-1 (NADPH oxidase subunit p47^phox^)-deficient neutrophils formed histone H3-containing web-like NET structure as readily as NCF-1-sufficient cells upon stimulation by unopsonized *C*. *albicans* (Fig 2A and 2B). Time-lapse live cell imaging showed that neutrophils underwent robust NETosis after encountering opsonized *C*. *albicans* (Fig 2C and S1 Video) whereas NET formation induced by unopsonized organism was less so (Fig 2C and S2 Video). Importantly, similar to stimulation of NCF-1-sufficient neutrophils with opsonized or unopsonized *C*. *albicans* (Fig 2C, S1 and S2 Videos), stimulation of NCF-1-deficient neutrophils by unopsonized *C*. *albicans* resulted in loss of lobular shape in the nucleus and chromatin decondensation (Fig 2C and S3 Video). These data demonstrate that NCF-1-deficient neutrophils underwent NETosis after *C*. *albicans* challenge. Consistently, *Ncf-1*^*-/-*^ and *Ncf-1*^*+/+*^ neutrophils releases comparable levels of dsDNA in response to stimulation by unopsonized *C*. *albicans*, whereas the response was greatly reduced in *Ncf-1*^*-/-*^ neutrophils upon challenge with opsonized organisms (Fig 2D). Additionally, treatment with MitoTEMPO (mitochondrial ROS inhibitor) did not affect NET formation in neutrophils stimulated by unopsonized *C*. *albicans* (Fig 2E). Our results indicate that unopsonized *C*. *albicans*-induced NET formation is independent of NADPH oxidase and mitochondrial ROS.

### Neutrophil killing of unopsonized *C*. *albicans* requires dectin-2-mediated NET formation

**Fig 3:**
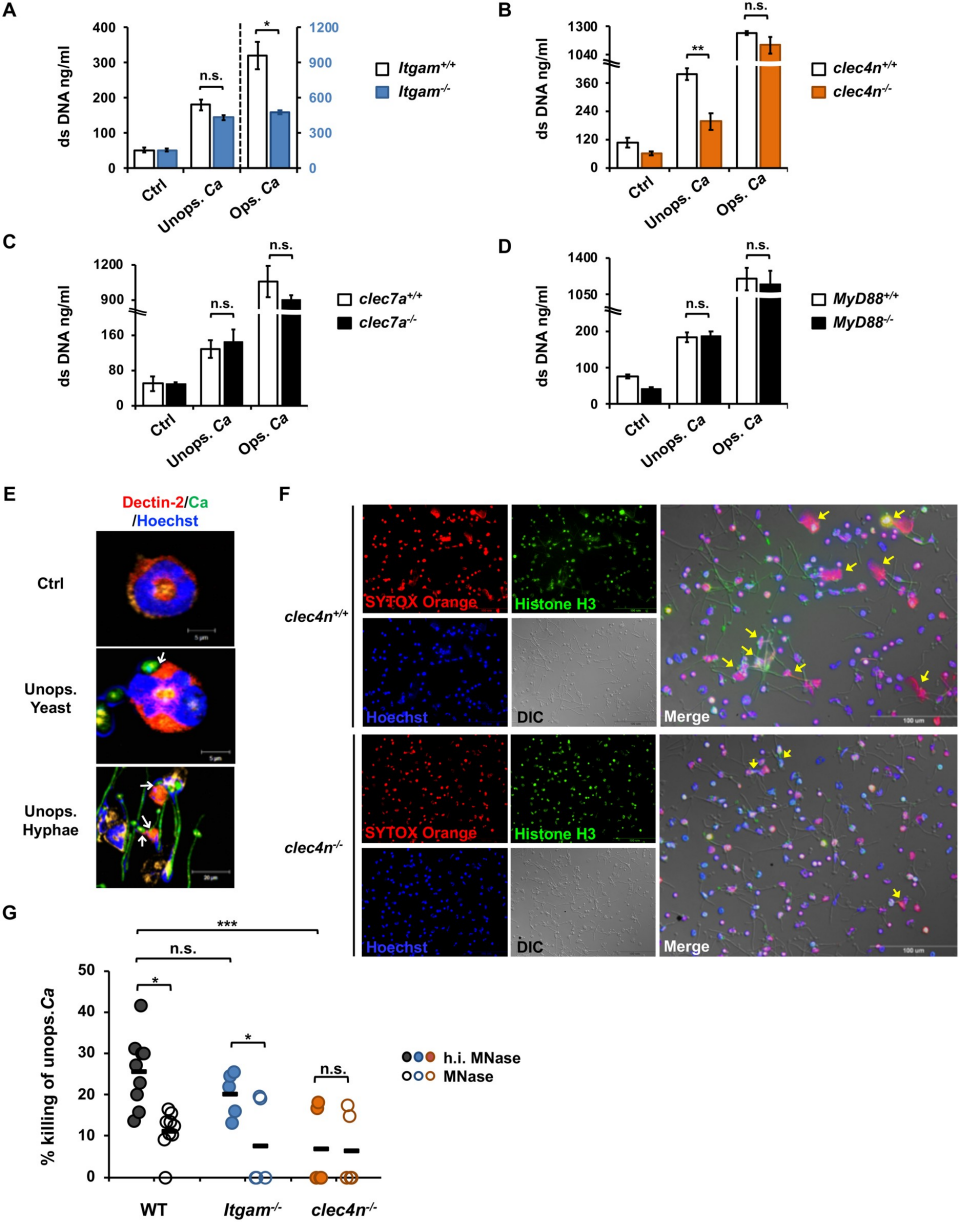
Neutrophil killing of unopsonized *C*. *albicans* requires dectin-2-mediated NET formation. WT, *Itgam*^*-/-*^ (A), *clec4n*^*-/-*^ (B), *clec7a*^*-/-*^ (C), and *MyD88*^*-/-*^ (D) neutrophils were stimulated or not (Ctrl) with opsonized (Ops. Ca) or unopsonized (Unops. Ca) *C*. *albicans* at MOI of 2 for 3 h. Extracellular DNA was quantified by Quant-iT PicoGreen dsDNA assay. (n = 3). All experiments were performed three times. Data from one representative experiment are shown and presented as mean ± SD. *, *p <* 0.05; **, *p <* 0.01; n.s., not significant, as analyzed by Student’s *t* test. (E) Neutrophils were stimulated with pre-germinated (Unops. Hyphae) or ungerminated (Unops. Yeast) GFP-expressing *C*. *albicans* OG1 (green). Cells were cytospun, permeabilized and stained for dectin-2 (red) and nucleus (blue). Arrows point to where dectin-2 contacts either the yeast or hyphal form of the fungus. (F) *Clec4n*^*+/+*^ and *clec4n*^*-/-*^ neutrophils were stimulated with unopsonized *C*. *albicans* at MOI of 2 for 3 h and stained with anti-histone H3 antibody (green), cell-impermeable DNA dye SYTOX Orange (red), cell-permeable DNA dye Hoechst 33258 (blue). Immunofluorescence images were viewed under fluorescence microscope. DIC, differential interference contrast image. Yellow arrows point to cells that undergo NETosis. (G) WT, *Itgam*^*-/-*^, and *clec4n*^*-/-*^ neutrophils were incubated with unopsonized *C*. *albicans* at MOI of 2 in HBSS supplemented with 10 U/ml of MNase or heat-inactivated MNase (h.i. MNase). Wells containing *C*. *albicans* only without neutrophils were used as control. Controls were incubated in medium containing h.i. MNase or MNase. Three hours after incubation, medium was collected and cold H_2_O (pH = 11) was added to lyse cells. *C*. *albicans* was detached by mini cell scraper and vigorous pipetting. The number of viable fungi was determined by plating the supernatant on yeast-peptone-dextrose agar plate. Colony counts (CFU) were enumerated 2–3 days later. The ability of neutrophils to kill *Candida* is presented as % killing of *C*. *albicans* which was calculated by dividing the difference of CFU counts between the control group (without neutrophils) and neutrophil-added groups with MNase or h.i. MNase treatment by the counts of respective control. WT, n = 9; *Itgam*^*-/-*^ and *clec4n*^*-/-*^, n = 5 each. Each n represents neutrophils collected from one mouse. Data were pooled from 3 independent experiments and presented as mean ± SD. *, *p <* 0.05; n.s., not significant, as analyzed by Student’s *t* test by comparing the 2 groups linked by a bracket.

We used receptor-deficient neutrophils to identify the receptors that mediate opsonized and unopsonized *C*. *albicans*-induced NETosis. Results showed that while CR3 deficiency (*Itgam*^*-/-*^) reduced dsDNA release triggered by opsonized *C*. *albicans* (Fig 3A), dectin-2 deficiency (*Clec4n*^*-/-*^) reduced that induced by unopsonized organism (Fig 3B). Neither dectin-1 nor MyD88 was involved in NETosis induced by either opsonized or unopsonized organism (Fig 3C and 3D). Confocal microscopic images showed that there was direct contact between dectin-2 and unopsonized *C*. *albicans*, whether it is in yeast or hyphal form (Fig 3E). Dectin-2 deficiency abrogated the formation of histone H3-containing NET structure after *C*. *albicans* challenge (Fig 3F). Thus, dectin-2 recognition of unopsonized *C*. *albicans* by neutrophils results in NETosis.

To study whether NET can kill *C*. *albicans*, we added DNA digestion enzyme, micrococcal nuclease (MNase) to the wells at the time when *C*. *albicans* was added. While the abilities of WT and CR3-deficient neutrophils to kill unopsonized *C*. *albicans* were comparable (WT: 25.8 ± 8.6%; CR3-deficient: 20.3 ± 5.5%), their killing functions were significantly diminished after MNase treatment (WT: 11.2 ± 4.8%; CR3-deficient: 7.7 ± 10.6%) (Fig 3G). It appears that neutrophil killing of unopsonized *C*. *albicans* is mediated by NET and independent of CR3 expression. Compared to WT and CR3-deficient cells, dectin-2-deficient neutrophils had lower ability to kill unopsonized *C*. *albicans* (6.5 ± 8.9%) (Fig 3G), yet such function was not affected by MNase treatment (7.0 ± 9.6%). These results together reveal that neutrophil killing of unopsonized *C*. *albicans* requires dectin-2-mediated NET formation.

### Dectin-2 mediates NET formation through Syk-Ca^2+^-PKCδ signaling pathway in response to unopsonized *C*. *albicans*

**Fig 4:**
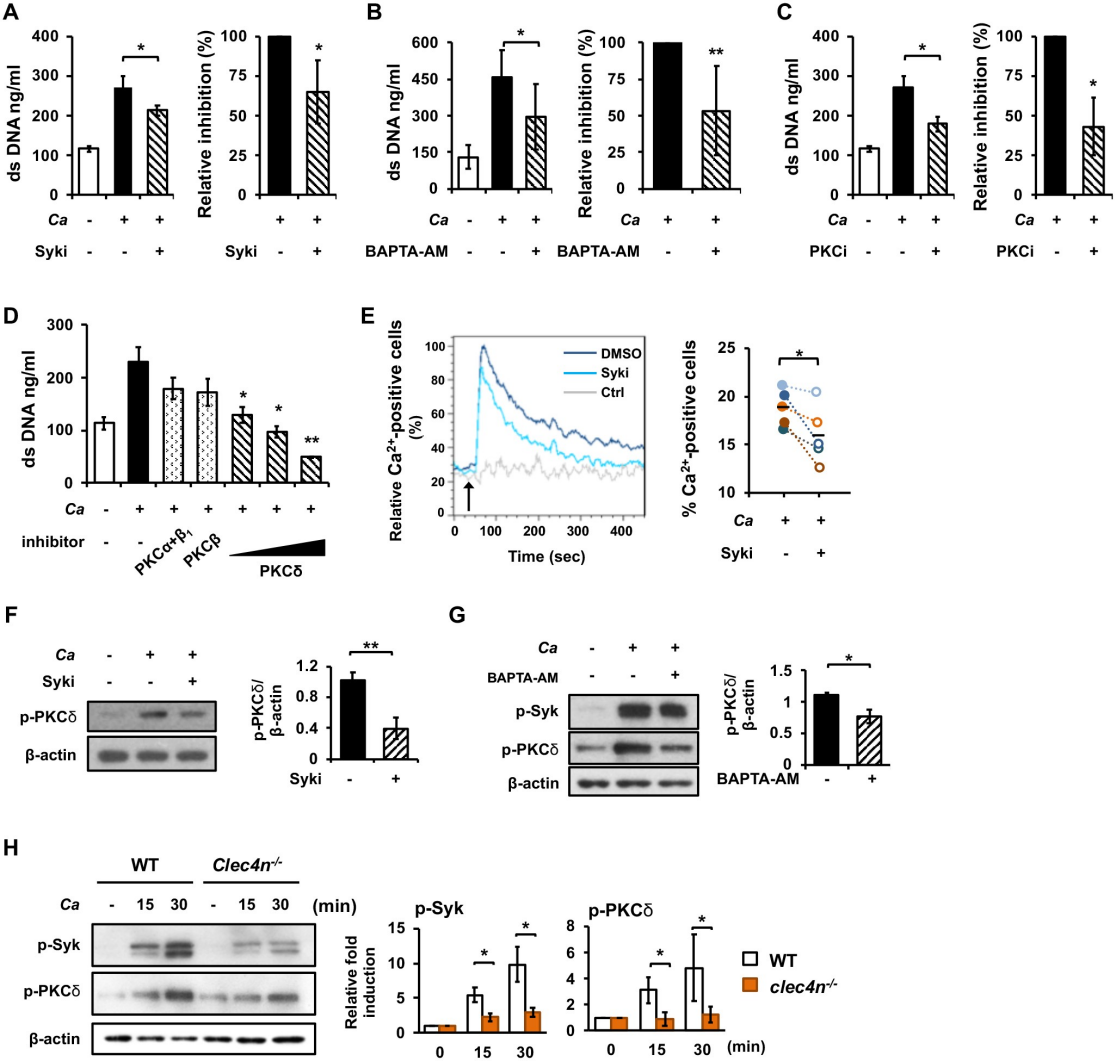
Unopsonized *C*. *albicans* induces NETosis through dectin-2-Syk-Ca^2+^-PKCδ pathway. WT (A-H) and *Clec4n*^*-/-*^ (H) neutrophils were stimulated with unopsonized *C*. *albicans*. (A-E) Cells were pre-treated (+) or not (-) with Syk inhibitor (Syki, 10 μM of SykI) (A), Ca^2+^ chelator (10 μM of BAPTA-AM) (B), PKC inhibitor (PKCi, 10 μM of Ro318220) (C), and inhibitor to PKC isoforms (250 μM of inhibitor to PKCα+β1, 2.5 μM of inhibitor to PKCβ, and 0.8, 4, 20 μM of inhibitor to PKCδ) (D) for 30 min before stimulation with (+) or without (-) unopsonized *C*. *albicans* at MOI of 2 for 3 h. Extracellular DNA was quantified by Quant-iT PicoGreen dsDNA assay. Relative inhibition (%) was calculated by dividing the value of inhibitor-treated group by that of the untreated (A-C). (E) Cells were first loaded with Ca^2+^ indicator and then pre-treated (Syki) or not (DMSO) with 10 μM of SykI for 30 min. After treatment, cells were stimulated (DMSO and Syki) or not (Ctrl) with unopsonized *C*. *albicans* at MOI of 4. Intracellular Ca^2+^ content was analyzed by flow cytometry from before stimulation until 450 sec after. Arrow points to the time when *C*. *albicans* was added. Maximal % of Ca^2+^-positive cells in unopsonized *C*. *albicans*-stimulated group was taken as 100% intensity (Max). The Ca^2+^ response of other time points was normalized against the maximal response and is shown as relative Ca^2+^-positive cells (%) (calculated under the kinetic mode of FlowJo software). Line graphs showing the kinetics of Ca^2+^ responses in the three groups were analyzed and overlaid by FlowJo software. Bar graph on the right shows % Ca^2+^-positive cells at maximal response (20–50 sec, gating strategy is shown in S1 Fig). (F, G) Cells were pre-treated with Syk inhibitor SykI (F) or Ca^2+^ chelator BAPTA-AM (G) before stimulation by pre-germinated unopsonized *C*. *albicans* at MOI of 2. At 30 min of stimulation, cell lysates were collected and subject to Western blot analysis for phosphorylated-Syk and -PKCδ. β-actin was used as loading control. Relative intensities of p-Syk and p-PKCδ are quantified by ImageJ and shown as bar graphs next to the blot. (H) WT and *clec4n*^*-/-*^ neutrophils were stimulated with pre-germinated unopsonized *C*. *albicans* at MOI of 2 for 15 and 30 min. Cell lysates were collected and subject to Western blot analysis for p-Syk and p-PKCδ as described in (G). Relative intensities of p-Syk and p-PKCδ in stimulated cells were normalized against their respective unstimulated controls and shown as relative fold induction. All Western blot experiments were performed three times. Data from one representative experiment are shown. Data are presented as mean ± standard error of the mean (SEM). *, *p <* 0.05; **, *p <* 0.01, as analyzed by Student’s *t* test comparing the 2 groups linked by a bracket.

We used pharmacological inhibitors to inhibit activation of signaling molecules and found that inhibition of Syk, Ca^2+^ influx, and PKCs significantly diminished NET formation (Fig 4A, 4B and 4C). While different isoforms of PKC family have their unique roles in modulating NETosis [24], our results showed that inhibition of PKCδ dose-dependently, but not PKCα and PKCβ, reduced the level of NETosis (Fig 4D). These results together indicate that Syk, Ca^2+^ influx, and PKCδ are involved in unopsonized *C*. *albicans*-induced NET. Furthermore, cells treated with Syk inhibitor had lower levels of Ca^2+^ influx, less Ca^2+^-positive cells (S1 Fig and Fig 4E) and lowered the level of phosphorylated PKCδ (Fig 4F). Cells treated with Ca^2+^ chelator had lower levels of phosphorylated PKCδ but not that of phosphorylated Syk (Fig 4G). In line with the observation that dectin-2 deficiency reduced the levels of phosphorylated Syk and PKCδ after stimulation (Fig 4H), our results clearly demonstrate that unopsonized *C*. *albicans* induces NETosis through dectin-2 and its downstream Syk-Ca^2+^-PKCδ pathway.

### NE nuclear translocation is involved in NCF-1-independent NETosis through Syk-Ca^2+^-PKCδ

**Fig 5:**
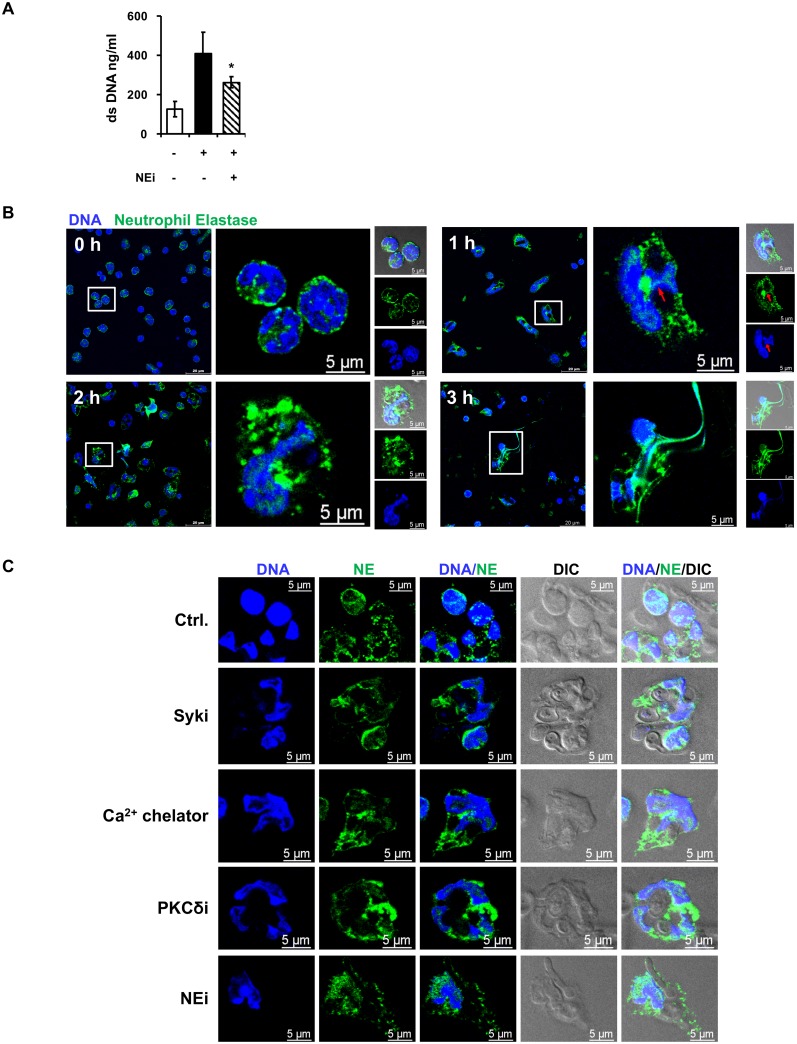
NE nuclear translocation is involved in NCF-1-independent NETosis through Syk-Ca^2+^-PKCδ. (A) Neutrophils were pre-treated (+) or not (-) with neutrophil elastase inhibitor (NEi, 10 μM of sivelestat) for 30 min before stimulation (+) or not (-) with unopsonized *C*. *albicans* at MOI of 2 for 3 h. Extracellular DNA was quantified by Quant-iT PicoGreen dsDNA assay. *, *p <* 0.05 as analyzed by Student’s *t* test comparing the groups treated with and without inhibitor. (B) Neutrophils were seeded on coverslips and stimulated with unopsonized *C*. *albicans* at MOI of 2. At indicated time after stimulation, cells were stained with anti-neutrophil elastase antibody (green) and cell-permeable DNA dye Hoechst 33258 (blue). Immunofluorescence images were viewed under confocal microscope. DIC, differential interference contrast image. Red arrow point to nuclear translocation of NE. (C) Cells were pre-treated with Syk inhibitor (10 μM of SykI, Syki), Ca2^+^ chelator (10 μM of BAPTA-AM), PKCδ inhibitor (20 μM of Rottlerin, PKCδi), and NE inhibitor (10 μM of sivelestat, NEi) before stimulation. Ctrl., cells incubated in HBSS containing 0.5% DMSO. At 2 h of stimulation, cells were stained with anti-neutrophil elastase antibody (green) and cell-permeable DNA dye Hoechst 33258 (blue). Immunofluorescence images were viewed under confocal microscope. DIC, differential interference contrast image.

Neutrophils treated with neutrophil elastase inhibitor sivelestat had reduced NET formation upon stimulation by unopsonized *C*. *albicans* (Fig 5A). Immunofluorescence images showed that NE was distributed in the cytoplasm and was separated from the nuclear region before stimulation (Fig 5B). Responding to unopsonized *C*. *albicans* challenge, NE aggregated into larger puncta and started translocating to the nucleus, especially to the decondensed area by 1 h of stimulation (Fig 5B). At 2 h after stimulation, the granules containing NE began to disintegrate, and NE was localized in the nucleus of cells that was ready for NETosis (2 h, Fig 5B). By 3 h after stimulation when NETotic structure began to form, NE was released to the extracellular space and bound to extracellular DNA fibers (3 h, Fig 5B). While nuclear translocation of NE occurred after stimulation with unopsonized *C*. *albicans* (Ctrl. in Fig 5C), neutrophils treated with pharmacological inhibitor to Syk, Ca^2+^ influx, PKCδ or NE exhibited condensed chromatin structure and their NE remained in the perimeter of the nucleus (Fig 5C), suggesting that Syk, Ca^2+^ influx, PKCδ and NE activity regulate chromatin decondensation as well as NE nuclear translocation. Together these data indicate that dectin-2 downstream signaling pathway mediates NE translocation. Importantly, NCF-1 deficiency did not affect NE nuclear translocation upon stimulation with unopsonized *C*. *albicans* (2 h, S2 Fig) although the number of NE-aggregated puncta was reduced (1 h, S2 Fig). NE was released along with DNA fibers in *Ncf-1*^*-/-*^ cells by 3 h after stimulation (S2 Fig). It appears that NCF-1 is not involved in NE nuclear translocation but may participate in granule aggregation after stimulation by unopsonized *C*. *albicans*.

### Unopsonized *C*. *albicans*-induced NET formation is dependent on PAD4 enzymatic activity

**Fig 6:**
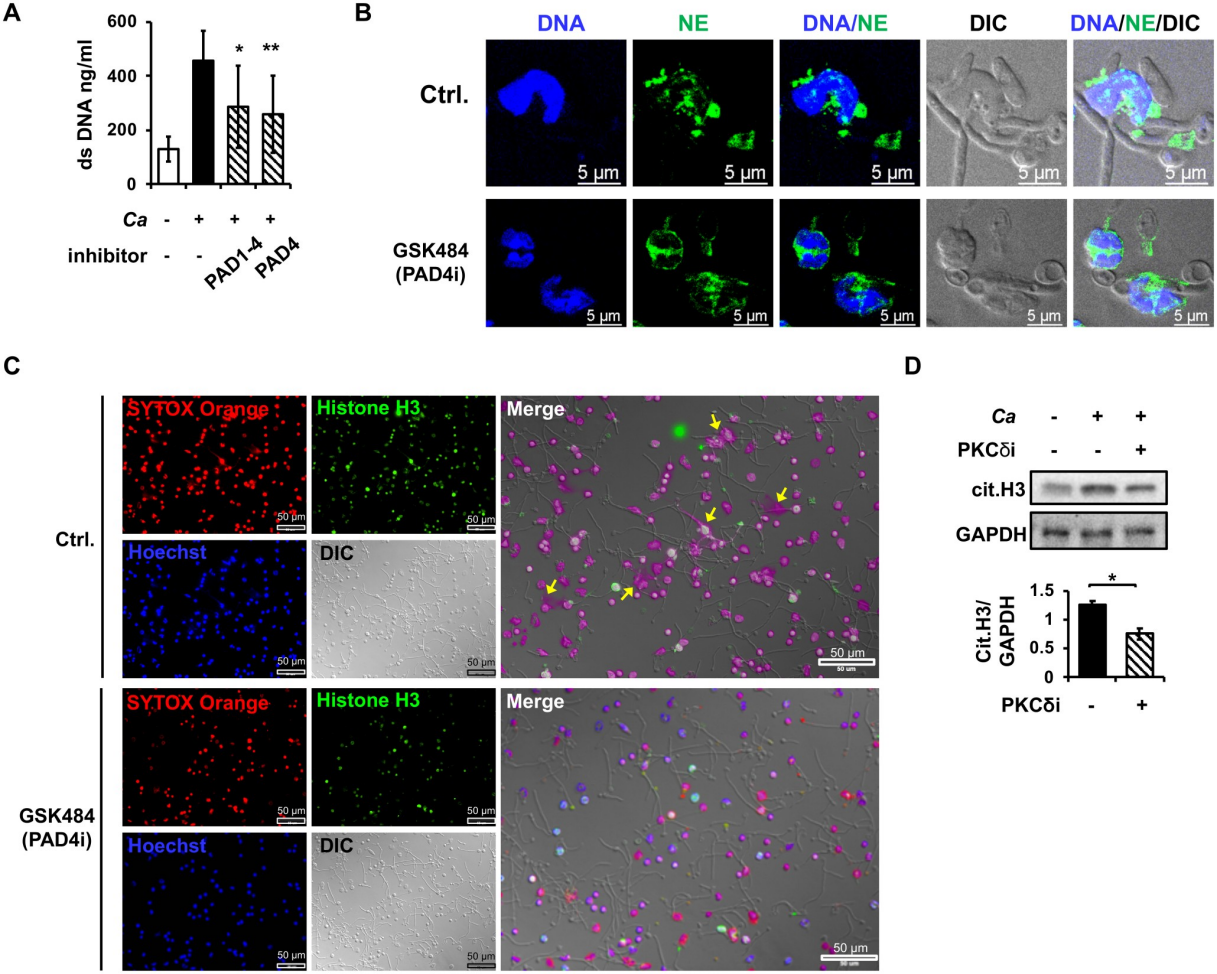
Unopsonized *C*. *albicans*-triggered NETosis is dependent on PAD4. (A) Neutrophils were pre-treated(+) or not (-) with PAD1-4 (10 μM of CC-Cl-amidine) or PAD4 (10 μM of GSK484) inhibitor for 30 min before stimulation (+) or not (-) with unopsonized *C*. *albicans* at MOI of 2 for 3 h. Extracellular DNA was quantified by Quant-iT PicoGreen dsDNA assay. (B) Cells were pre-treated with PAD4 inhibitor (PAD4i, 10 μM of GSK484) for 30 min before stimulation. At 2 h of stimulation, cells were stained with anti-neutrophil elastase antibody (green) and cell-permeable DNA dye Hoechst 33258 (blue). Immunofluorescence images were viewed under confocal microscope. DIC, differential interference contrast image. Ctrl., cells incubated in HBSS containing 0.1% DMSO. (C) Neutrophils pretreated or not (Ctrl) with PAD4 inhibitor (10 μM of GSK484, PAD4i) were stimulated with unopsonized *C*. *albicans* at MOI of 2 for 3 h. Cells were stained with anti-histone H3 antibody (green), cell-impermeable DNA dye SYTOX Orange (red), cell-permeable DNA dye Hoechst 33258 (blue). Immunofluorescence images were viewed under fluorescence microscope. DIC, differential interference contrast image. Arrows point to H3-containing web-like structure. (D) Cells were pre-treated with PKCδ inhibitor (PKCδI, 20 μM of Rottlerin) before stimulation with *C*. *albicans*. At 30 min of stimulation, cell lysates were collected and subject to Western blot analysis for citrullinated-histone H3 (cit.H3). GAPDH was used as a loading control. The experiment was performed 3 times. Data from one representative experiment are shown. Relative intensities of cit.H3 against GAPDH are shown below the blot. Data are presented as mean ± standard error of the mean (SEM).*, *p <* 0.05; **, *p <* 0.01, as analyzed by Student’s *t* test comparing the two groups treated with and without inhibitor (A, D).

Treatment with inhibitor to PAD1-4 or PAD4 reduced unopsonized *C*. *albicans*-induced NET formation (Fig 6A). PAD4 inhibitor also impeded NE nuclear translocation and chromatin decondensation (Fig 6B) as well as the formation of histone H3-containing web-like NET structure (Fig 6C). Thus, PAD4 activity is required for unopsonized *C*. *albicans*-induced nucleus decondensation and NE nuclear translocation. Interestingly, inhibition of PKCδ reduced the level of citrullinated H3 (Fig 6D). Since histone H3 citrullination is catalyzed by PAD4 [14], these results show that recognition of unopsonized *C*. *albicans* by dectin-2 leads PAD4-dependent NET formation.

### NET formation in peritoneal cavity after *C*. *albicans* infection

**Fig 7:**
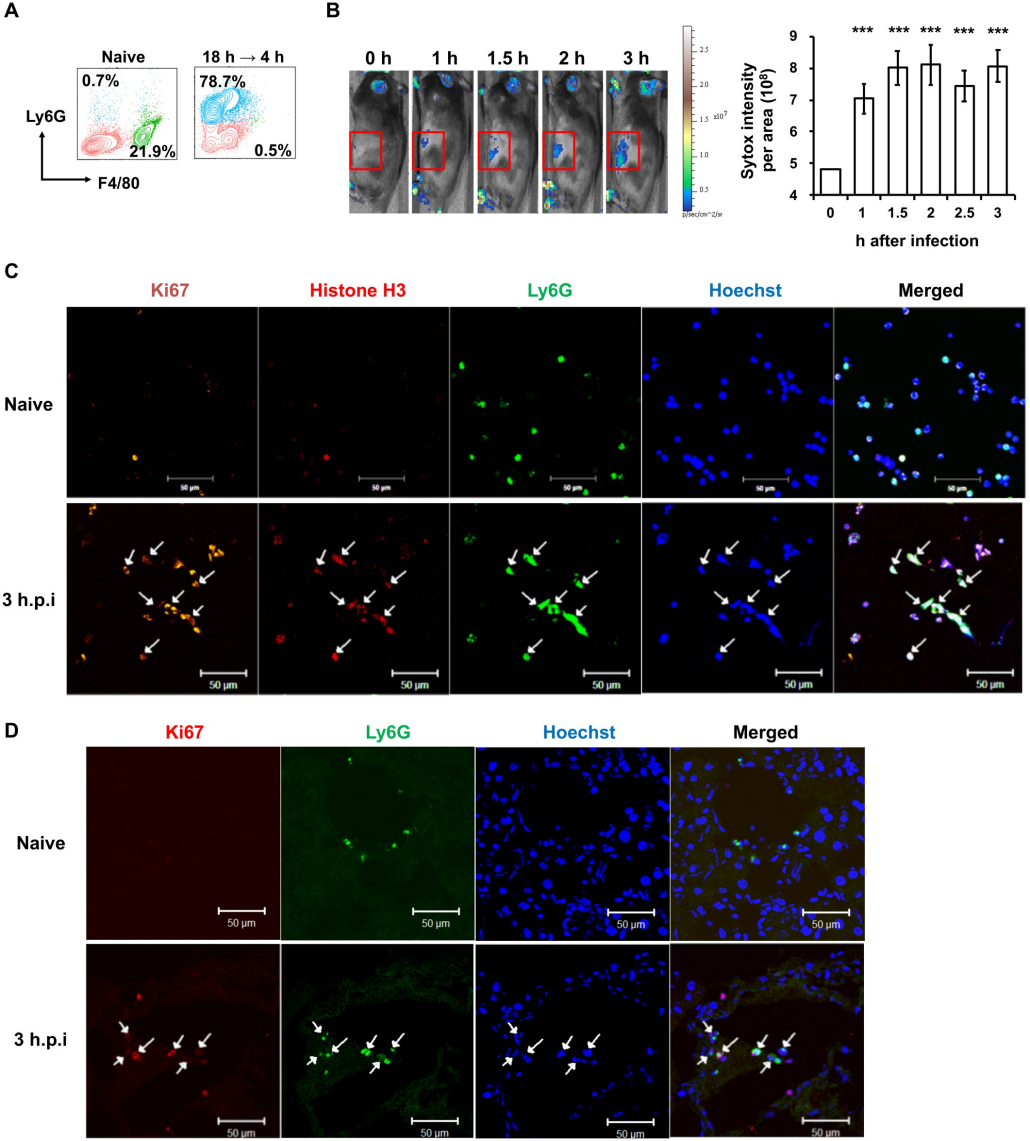
*C*. *albicans* infection triggers NETosis in mice. (A) Peritoneal exudates were harvested from naïve mice and from mice at 4 h after receiving two peritoneal injections of 9% casein (18 h apart (18 h → 4 h). Cells were stained with anti-Ly6G and anti-F4/80 antibodies and subject to flow cytometric analysis. Contour plots show the % of Ly6G^+^F4/80^-^ neutrophils (blue population) and Ly6G^-^F4/80^+^ macrophages (green population) among total cells. (B-D) WT mice were given peritoneal injections of casein as described above. At 4 h after the second casein injection, mice received 1 × 10^8^ of *C*. *albicans* intraperitoneally. (B) Mice were injected with SYTOX Orange intraperitoneally at the same time when *C*. *albicans* strain SC5314 was administered. Mice were imaged on the side by IVIS (Ex/Em = 570/620) to record SYTOX Orange signals for 3 h starting at the time when *C*. *albicans* was administered. Photons in user-specified region of interest (ROI, gated area) was measured by Living Image 3.2 software. Relative intensity of total photons in ROI at each time point was calculated based on the intensity at 1 h after infection. n = 10. Data are presented as mean ± SD. ***, *p <* 0.005, as analyzed by Student’s *t* test comparing the intensity at each time point to that at 1 h after infection. (C) Three hours after *C*. *albicans* infection, peritoneal exudates were collected and seeded on coverslips. Cells on the coverslips were permeabilized and stained for Ki67 (orange), histone H3 (red), Ly6G (green) and nucleus (blue) and viewed under fluorescence microscope. Arrows point to Ly6G^+^Ki67^+^ cells. (D) Three hours after *C*. *albicans* infection, mesenteric tissues were collected and embedded in O.C.T. Cryosections were stained for Ki67 (red), Ly6G (green) and nucleus (blue) and viewed under confocal microscope. Arrows point to Ly6G^+^Ki67^+^ cells.

A *C*. *albicans* peritonitis model was established to study the role of NET formation *in vivo*. Since there are few neutrophils in the peritoneal cavity of normal mice, we gave mice two intraperitoneal injections of casein 18 h apart to enrich the neutrophil population. Mice were given *C*. *albicans* yeasts intraperitoneally 4 h after the second injection of casein when peritoneal neutrophils constituted about 78.8% of the whole peritoneal cell population (Fig 7A). In vivo imaging system (IVIS) spectrum images showed that *C*. *albicans* infection induced release of extracellular DNA into peritoneal cavity as early as 1.5 h after infection and remained at relatively the same level until 3 h later (Fig 7B). Web-like DNA structures that were positive for Ki67 (a novel marker for mature neutrophils that undergo NETosis [13]), histone H3, and Ly6G cells were observed in peritoneal exudates from mice given *C*. *albicans* (Fig 7C). Cells on the mesenteric tissues collected from infected mice also stained positive for Ki67 and Ly6G (Fig 7D). In the peritonitis candidiasis model, we observed NET formation in the peritoneal cavity and NETotic cells on the mesenteric tissues.

### NCF-1-independent NETosis restrains *C*. *albicans* spread from peritoneal cavity to kidney

**Fig 8:**
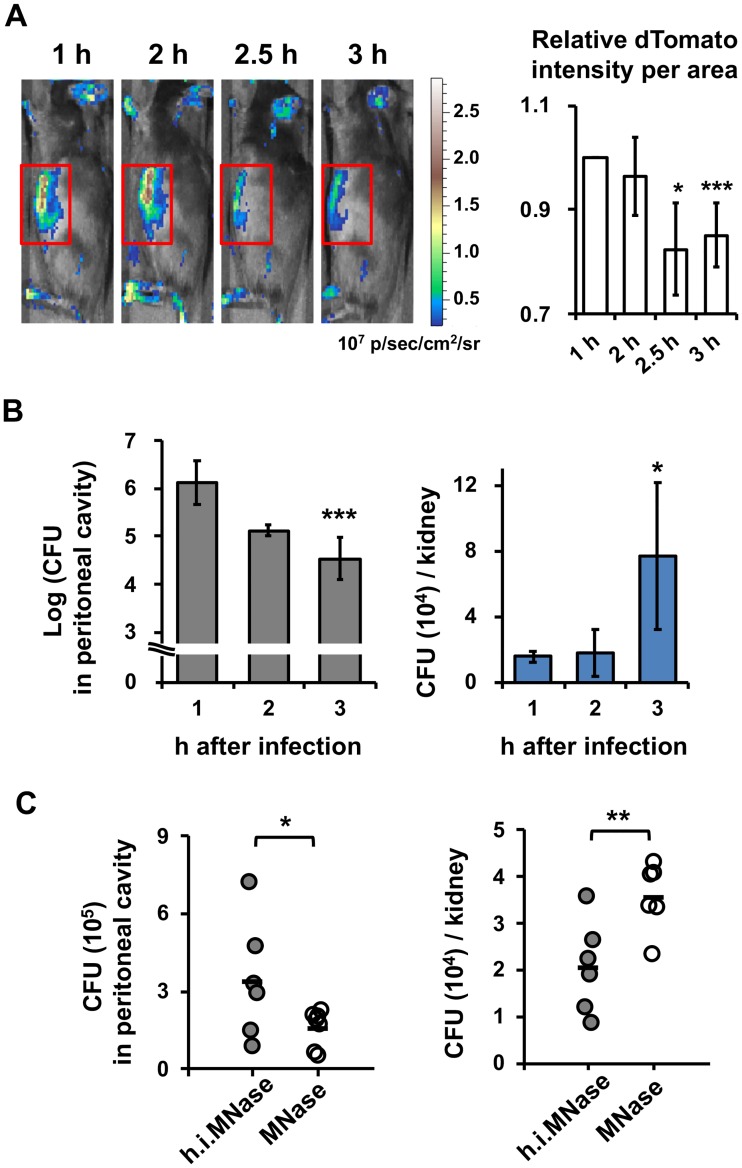
NET formation reduces *C*. *albicans* spread from peritoneal cavity to kidney. WT mice were injected with 9% casein intraperitoneally as described above. (A) At 4 h after the second injection, mice were injected with 3 × 10^8^ of dTomato-expressing *C*. *albicans* strain CAF2 intraperitoneally and imaged on the side by IVIS (Ex/Em = 570/620). dTomato signals were recorded for 3 h starting 1 h after *C*. *albicans* administration. Photons in ROI (gated area) was measured by Living Image 3.2 software. Relative intensity of total photons in ROI was calculated based on the intensity at 1 h after infection. n = 5. Data are presented as mean ± SD. (B) Mice were injected with 1 × 10^8^ of *C*. *albicans* strain SC 5314 intraperitoneally at 4 h after second casein injection. At indicated times after infection, peritoneal fluid and kidneys were collected. Fungal burdens were determined by plating. n = 4. (C) Mice were injected with 100 U of MNase or heat-inactivated MNase (h.i. MNase, in otherwise equivalent amount) intraperitoneally at the time of infection with 1×10^8^ of *C*. *albicans*. At 3 h after infection, peritoneal fluid and kidneys were collected. Fungal burdens were determined by plating. n = 6. *, *p <* 0.05; **, *p <* 0.01; ***, *p <* 0.005, as analyzed by Student’s *t* test comparing indicated time points to 1 h after infection (A, B) or the two groups linked by a bracket (C).

To monitor *C*. *albicans* spread, we infected mice with dTomato-expressing *C*. *albicans* when neutrophils were enriched in the peritoneal cavity. IVIS images showed that the intensity of fluorescence in the peritoneal cavity remained at relatively the same level at 1 and 2 h after infection and decreased thereafter (Fig 8A). Coinciding with decrease in intensity of dTomato, fungal burden in the peritoneal cavity also decreased by 3 h after infection and in the meantime, it was increased in the kidney (Fig 8B). We then treated mice with NET digestion enzyme micrococcal nuclease (MNase) or its heat-inactivated form (h.i. MNase) intraperitoneally and discovered that treatment with MNase compared to h.i. MNase decreased fungal burden in the peritoneal cavity [from (3.4 ± 2.3) × 10^5^ to (1.6 ± 0.7) × 10^5^ CFU] and increased that in the kidney [from (2.1 ± 1.0) × 10^4^ to (3.6 ± 0.7) × 10^4^ CFU/kidney) (Fig 8C). These results demonstrate that NET functions to restrain *C*. *albicans* in the peritoneal cavity and keep it from spread to the kidney. In the meanwhile, *Ncf-1*^*-/-*^ mice were infected by *C*. *albicans* intraperitoneally to assess whether NCF-1 participates in inducing NETosis in vivo. Data in S3A and S3B Fig showed that NCF-1 deficiency did not affect NET formation in peritoneal exudate nor did it affect the Ki67^+^Ly6G^+^ population in mesenteric tissues, indicating that an NCF-1-independent NETosis response to *C*. *albicans* occurs in vivo. Interestingly, however, *Ncf-1*^*-/-*^ mice had significantly higher fungal burden in the peritoneal cavity but a comparable level in the kidney compared to *Ncf-1*^*+/+*^ mice at 3 h after infection (S3C Fig). Since ROS is important to phagocytic cell clearance of *C*. *albicans* [18], these results indicate that unopsonized *C*. *albicans*-induced NCF-1-independent NETosis that restricts fungal spread does occur in vivo, but fungal clearance involves more than just NETosis.

### PAD4 is important to prevent fungal spread

**Fig 9:**
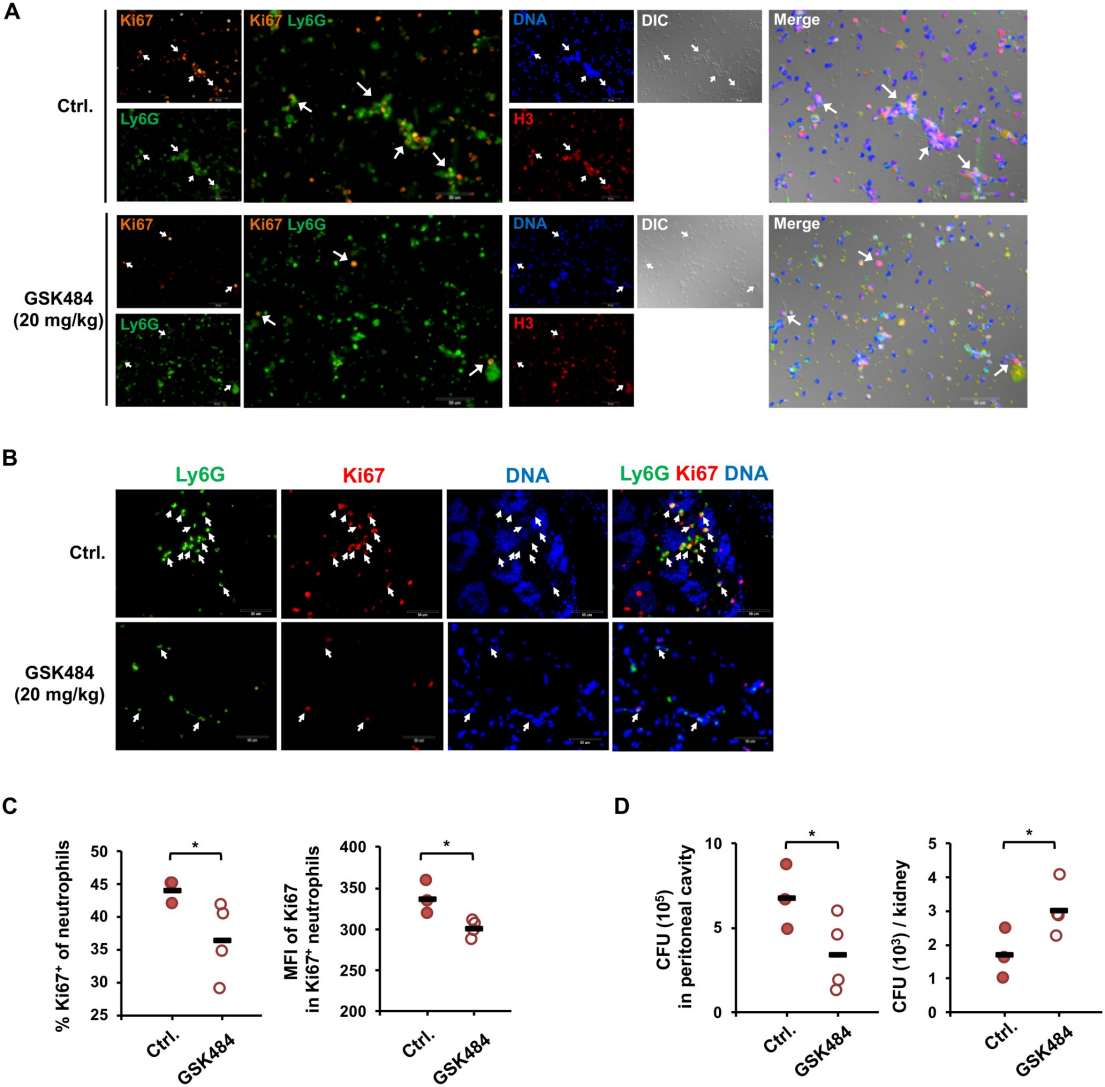
GSK treatment impedes NET formation and promotes *C*. *albicans* spread from peritoneal cavity to kidney. (A) WT mice were given two injections of 9% casein as described above. At 4 h after the second injection, mice were injected with GSK484 (20 mg/Kg) or HBSS containing 10% of DMSO intraperitoneally (Ctrl.) at the time of challenge with 1 × 10^8^ *C*. *albicans*. At 3 h after infection, peritoneal exudates, mesenteric tissues and kidneys were collected. (A) Peritoneal exudates were seeded on coverslips and incubated for 1 h. Cells were permeabilized and stained for Ki67 (orange), histone H3 (red), Ly6G (green) and nucleus (blue) and viewed under fluorescence microscope. DIC, differential interference contrast image. Arrows point to Ly6G^+^Ki67^+^ cells. (B) Mesenteric tissues were collected and embedded in O.C.T. Cryosections were stained for Ki67 (red), Ly6G (green) and nucleus (blue) and viewed under fluorescence microscope. (C) Peritoneal exudates were stained with anti-CD11b, -Ly6G and -Ki67antibodies and subject to flow cytometric analysis. The percentages of Ki67^+^ cells among total neutrophils (CD11b^+^Ly6G^+^) population are shown as % Ki67^+^ of neutrophils. The mean fluorescence intensity (MFI) of Ki67 represents the level of Ki67 expression in Ki67^+^ cells. Gating strategy for Ki67 was illustrated in S4B Fig. Data were pooled from two independent experiments. (D) Fungal counts in total peritoneal fluid and kidney homogenates were determined by plating. Fungal colonies were counted 2–3 days later. Data were pooled from 4 independent experiments. *, *p <* 0.05; ***, *p <* 0.005, as analyzed by Student’s *t* test.

Our data in Fig 6 showed that PAD4 is involved in NE nuclear translocation and inducing NET formation in vitro. To explore the role of PAD4 in *C*. *albicans* infection, we treated mice with PAD4 inhibitor GSK484. Mice were given GSK484 before intraperitoneal injection of *C*. *albicans*. Results showed that inhibition of PAD4 reduced the formation of web-like structure and Ki67 expression in peritoneal Ly6G^+^ cells and Ki67^+^Ly6G^+^ cell population in mesenteric tissues (Fig 9A and 9B). Flow cytometric analysis also revealed that GSK484 treatment reduced the percentage and the level of Ki67 in peritoneal infiltrating neutrophils (Fig 9C). In addition, GSK484 treatment decreased fungal CFU in the peritoneal cavity and increased that in the kidney by 3 h after infection (Fig 9D). Results of our in vitro (Fig 6) and in vivo studies together demonstrated that PAD4 regulates NETosis response to *C*. *albicans*.

### *C*. *albicans*-induced NET formation is dectin-2-dependent

**Fig 10:**
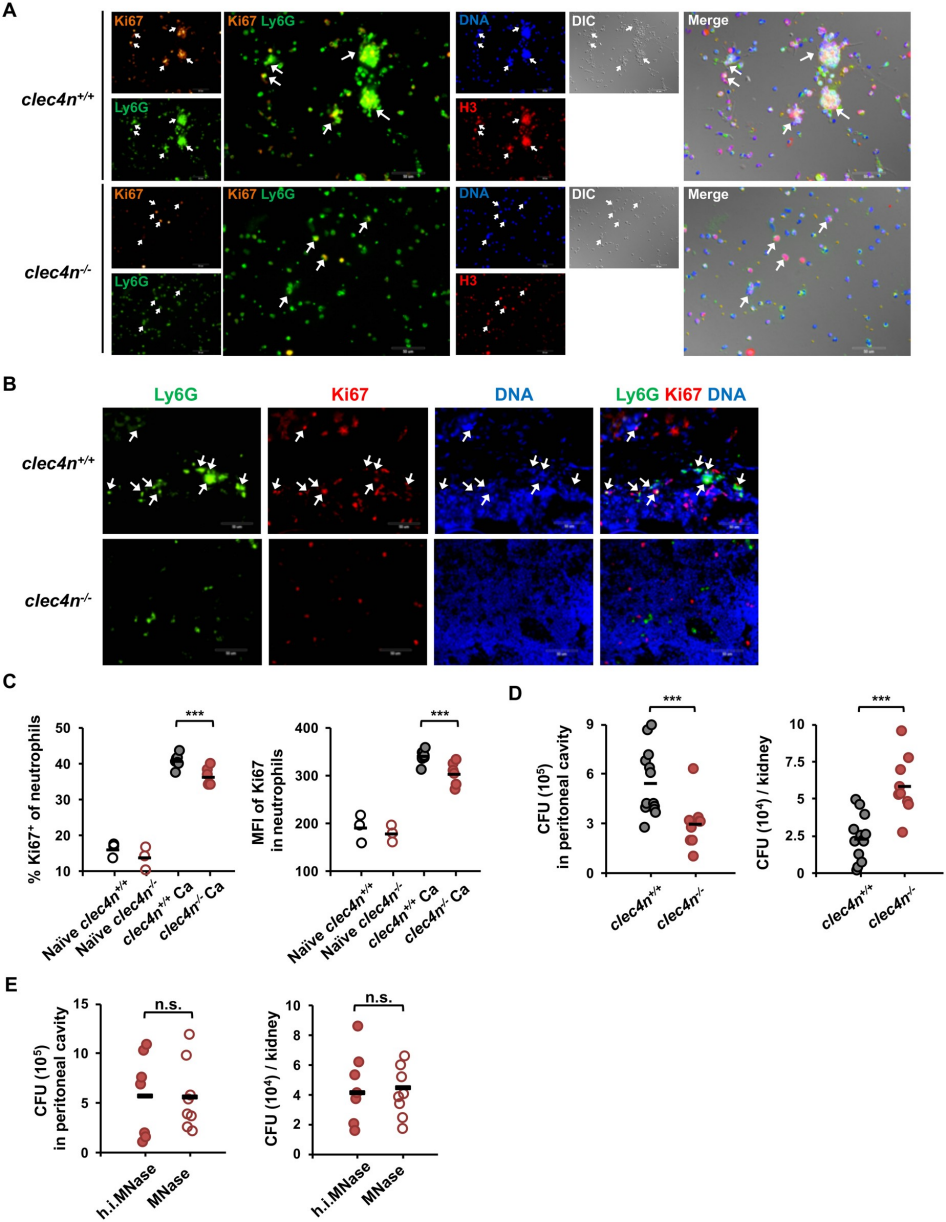
Dectin-2 deficiency reduces *C*. *albicans*-induced NETosis and increases fungal spread from peritoneal cavity to kidney. (A-C) *Clec4n*^*+/+*^ and *clec4n*^*-/-*^ mice were given two injections of 9% casein as described above. At 4 h after the second injection, mice were injected with or without 1 × 10^8^ of *C*. *albicans* intraperitoneally. At 3 h after infection, peritoneal exudates, mesenteric tissues and kidneys were collected. (A) Peritoneal exudates were seeded on coverslips and incubated for 1 h. Cells were permeabilized and stained for Ki67 (orange), histone H3 (red), Ly6G (green) and nucleus (blue) and viewed under fluorescence microscope. Arrows point to Ly6G^+^Ki67^+^ cells. (B) Mesenteric tissues were collected and embedded in O.C.T. Cryosections were stained for Ki67 (red), Ly6G (green) and nucleus (blue) and viewed under fluorescence microscope. DIC, differential interference contrast image. (C) Peritoneal exudates were stained with anti-CD11b, -Ly6G and -Ki67 antibodies and subject to flow cytometric analysis. The percentages of Ki67^+^ cells among total neutrophils (CD11b^+^Ly6G^+^) population are shown as % Ki67^+^ of neutrophils. The mean fluorescence intensity (MFI) of Ki67 represents the level of Ki67 expression in Ki67^+^ cells. Data were pooled from 2 independent experiments. Gating strategy for Ki67 was illustrated in S4B Fig. Naïve mice are uninfected mice receiving casein injection only. (D) Fungal counts in total peritoneal fluid and kidney homogenates were determined by plating. Fungal colonies were counted 2–3 days later. (*Clec4n*^*+/+*^ n = 13; *Clec4n*^*-/-*^ n = 9). Data were pooled from four independent experiments. ***, *p <* 0.005, as analyzed by Student’s *t* test. (E) *Clec4n*^*-/-*^ mice were given two injections of 9% casein as described above. Four hours after the second injection, mice were injected with 100 U of MNase or heat-inactivated MNase (h.i. MNase, in otherwise equivalent amount) intraperitoneally at the time when 1×10^8^ of *C*. *albicans* was administered. At 3 h after infection, peritoneal exudates and kidneys were harvest. Fungal counts in total peritoneal fluid and kidney homogenates were determined by plating. Fungal colonies were counted 2–3 days later. (n = 7–8). Data were pooled from 2 independent experiments. n.s., not significant, as analyzed by Student’s *t* test.

We then determined whether dectin-2 is involved in NETotic response to *C*. *albicans* in mice. Peritoneal neutrophil-enriched WT and dectin-2-deficient mice were intraperitoneally infected with *C*. *albicans*. While dectin-2 deficiency did not affect neutrophil recruitment to the peritoneal cavity (S4A Fig), peritoneal infiltrating neutrophils from infected dectin-2-deficient mice had less Ki67^+^Histone H3^+^ web-like structures than that from sufficient mice (Fig 10A). Compared to sufficient mice, dectin-2-deficient mice had significantly less Ki67^+^Ly6G^+^ population in mesenteric tissues (Fig 10B) and reduced Ki67 expression in neutrophils (Fig 10C) after *C*. *albicans* infection. Furthermore, dectin-2-deficient mice had significantly lower fungal burdens in the peritoneal cavity [(3 ± 1.5) × 10^5^ CFU] but greater burdens in kidneys [(5.9 ± 2.0) × 10^4^ CFU/kidney] than WT mice [(5.5 × ± 2.0) × 10^5^ CFU] in peritoneal cavity; (2.4 ± 1.5) × 10^4^ CFU/kidney] (Fig 10D). Digesting extracellular DNA by MNase did not affect the fungal burdens in the peritoneal cavity and kidney in dectin-2-deficient mice (Fig 10E), supporting the notion that the function of NET in restraining fungal spreading is through dectin-2. These results demonstrate the importance of dectin-2-mediated NETosis in keeping *C*. *albicans* from spreading to the kidney.

## Discussion

Opsonized *C*. *albicans*, through interaction with CR3, activates downstream Syk-dependent NADPH oxidase activation [18] and NADPH oxidase is required for opsonized *C*. *albicans*-induced NET formation [11]. Unlike NET formation induced by lipopolysacchride, phorbol 12-myristate 13-acetate (PMA) and *Shigella flexneri*, PAD4 has been reported not to be involved in opsonized *C*. *albicans*-induced NET formation in human and mouse [17, 23, 25]. Opsonized *C*. *albicans*-induced NET is inhibited by PKC inhibitor, but not Ca^2+^ chelator [17]. Thus, it appears that CR3 recognition of opsonized *C*. *albicans* sends signals to activate NADPH oxidase-dependent NET formation through Syk-PKC-ROS cascade, but PAD4 and Ca^2+^ do not take part in NET formation. Our study employing confocal microscopy, fluorescence microscopy, transmission electron microscopy, live cell imaging and PicoGreen dsDNA assay shows that unopsonized *C*. *albicans* triggers neutrophils to undergo a NADPH oxidase-independent NETosis. Distinct from opsonized organisms, unopsonized *C*. *albicans*-induced NET is through ligation of dectin-2 that drives Syk-Ca^2+^-PKCδ-NE/PAD4 signaling pathway. Our study together with those of others reveal that opsonized and unopsonized *C*. *albicans* utilize different receptors and different signaling pathways to trigger NETosis.

Employing PAD4^-/-^ and WT neutrophils Guiducci et al. showed in their recent publication [23] that in response to opsonized *C*. *albicans*, PAD4 deficiency reduced histone H3 citrullination (confocal microscopy of neutrophils in vitro). However, they found that PAD4 was not required for opsonized *C*. *albicans*-induced NETosis (quantified by SytoxGreen assay, immunofluorescence staining for confocal microscopic imaging and electron microscopic imaging). We showed that GSK484 treatment of neutrophils challenged with unopsonized *C*. *albicans* reduced NE translocation (immunofluorescence staining for confocal microscopic imaging), Sytox Organge^+^histone H3^+^ web-like structure formation (immunofluorescence staining for fluorescence microscopic imaging) and NETosis (quantified by Quant-iT PicoGreen dsDNA assay). Therefore, it appears that unlike challenge with opsonized *C*. *albicans*, PAD4 activation results in NETosis when neutrophils are challenged with unopsonized organisms in vitro. Furthermore, it is shown that PAD4 deficiency increased fungal CFU in kidney on day 3 and 7 after intravenous infection, decreased that in the tongue on day 1 after sublingual infection but no other time points [23]. We treated mice with PAD4 inhibitor GSK484 before intraperitoneally infected them with *C*. *albicans*. Inhibition of PAD4 reduced Ki67 expression and web-like structure formation in Ly6G^+^ cells in peritoneal exudate and Ki67^+^Ly6G^+^ cell population in mesenteric tissues (intracellular Ki67 straining followed by flow cytometric analysis and immunofluorescence staining for fluorescence microscopic imaging). In addition, GSK484 treatment decreased fungal CFU in the peritoneal cavity and increased that in the kidney at 3 h after infection. These data clearly demonstrated that PAD4 is important to *C*. *albicans*-induced NETosis in vivo. Regarding the role of PAD4 in host defense, our work with peritonitis infection together with that reported by Guiducci et al. with sublingual infection show that PAD4 functions to restrain fungal spread from the inoculation site to distal site during early phase of *C*. *albicans* infection [23]. We speculate that since NETosis-mediated restrain of fungal spread [16, 26] does not affect eventual fungal clearance, PAD4 is not required for control of fungal infection.

C-type lectin receptor engagement elicits proinflammatory cytokine response to stimulation by fungal ligand through Syk-mediated PKCδ activation [27]. PKCδ activity modulates CARD9/Malt1/Bcl10 signalosome formation to facilitate downstream NFκB translocation and subsequent cytokine production [27]. Deletion of *Prkcd*, but not *Prkca* nor *Prkcb* genes, abolishes TNF, IL-6 and IL-1β production by dendritic cells upon zymosan, curdlan or *C*. *albicans* stimulation [27], indicating the unique role of PKCδ in fungal challenge. We use pharmacological inhibitors for different PKC isoforms and uncover the importance of dectin-2 downstream PKCδ in histone H3 citrullination and NETosis in response to unopsonized *C*. *albicans*. Conventional PKCs are known to mediate NADPH oxidase-dependent ROS-mediated NETosis [16]. Our results reveal a PKCδ (novel PKC isoform)-mediated signaling pathway that is involved in unopsonized *C*. *albicans*-induced NADPH oxidase-independent NETosis. Our finding also suggests that CARD9/Malt1/Bcl10 signalosome which is downstream of PKCδ may function to mediate NE translocation and PAD4 activation, histone H3 citrullination and trigger NETosis.

C-type lectin dectin-1 has been reported to interfere with *C*. *albicans*-induced NET formation in human neutrophils through promoting phagocytosis [20]. NE is normally associated with granule membrane [28]. Upon phagocytosis, it is delivered to phagosome and sequestered within *C*. *albicans*-containing phagosome [20]. After which, its access to decondensed chromatin is blocked [20]. Metzler et al. observed in human neutrophils that NE is dissociated from granule membrane via ROS production to gain access to decondensed chromatin [28]. *C*. *albicans* yeast is known to be taken up and cleared by human neutrophils and the neutrophils remain intact [29]. Mouse neutrophils, however, allow engulfed *C*. *albicans* to germinate, resulting in membrane rupture and eventual cell death [29]. Our data show that dectin-1 and NADPH oxidase are not involved in NETotic response to unopsonized *C*. *albicans*. We also found that inhibiting NE reduces unopsonized *C*. *albicans*-induced NET formation, and that NE translocates to the nucleus is independent of NCF-1. We further demonstrated that Syk-Ca^2+^-PKCδ-PAD4 pathway modulates NE nuclear translocation and its access to decondensed chromatin without the involvement of ROS. Thus, it appears that chromatin decondensation and NE translocation as a result of dectin-2 downstream Syk-Ca^2+^-PKCδ-PAD4 signaling pathway are important to NADPH oxidase-independent NETosis.

Urban et al. employed pulmonary and subcutaneous *C*. *albicans* infection models to investigate NET formation [30]. In vivo NET formation in infected tissues was identified as structures that stained positive for cell-impermeable DNA dye (pre-injected before animals were killed), myeloperoxidase and histone. NET formation in subcutaneous tissue was observed on 6 days after subcutaneous infection and in the lungs at 24 h after intranasal infection [30]. NADPH oxidase deficiency greatly reduces NET formation in pulmonary *A*. *fumigatus* infection [31]. It appears that NADPH oxidase is involved in host NETosis response to pulmonary fungal infection in vivo. Ki67 is recently established as a NETosis marker [13]. Neutrophils undergoing NETosis in the lungs of *C*. *albicans*-infected mice as well as that in the brain of fungus-infected humans are Ki67-positive [13]. We enriched neutrophils in the peritoneal cavity of a mouse by injecting casein twice before *C*. *albicans* infection. Abundant peritoneal neutrophils allow a robust NET response to *C*. *albicans* infection. IVIS imaging showed extracellular DNA release as early as 1.5 h after infection and histone H3^+^Ki67^+^Ly6G^+^ neutrophils with web-like structures were observed in peritoneal exudates. The % of Ly6G^+^ neutrophils undergoing NETosis was quantified by flow cytometric analysis of intracellular Ki67. This *C*. *albicans* peritonitis model where neutrophils could be easily obtained without tissue homogenization and enzyme digestion is useful in quantifying NET and studying their function.

The role of dectin-2 in host defense against *C*. *albicans* is well documented. Dendritic cells utilize dectin-2 to recognize *C*. *albicans* for IL-6, IL-1β, and IL-23 production [32]. Dectin-2 signaling induces cytokine production through Syk-CARD9-NF-κB pathway [32]. While dectin-2-deficient dendritic cells have greatly reduced ability to produce cytokines in response to fungal α-mannan, dectin-2 deficiency also dampens host cytokine response to *C*. *albicans* [32]. Systemic *C*. *albicans* infection results in higher mortality in dectin-2-deficient mice than WT mice [32]. Neutrophils are major effector cells that kill *C*. *albicans* [5]. Whether dectin-2 plays a role in neutrophil response to *C*. *albicans* is an interesting question. It is reported that the role of dectin-2 in neutrophil ROS response to opsonized *C*. *albicans* is only marginal [19]. Our results show that dectin-2 recognizes unopsonized *C*. *albicans* for NETotic response, and dectin-2 deficient mice have reduced ability to restrain fungal spread from peritoneal cavity to kidney. These results suggest that *C*. *albicans* injected to the peritoneal cavity remain unopsonized at least for a short period of time to be recognized by dectin-2 and reveal a new role for dectin-2 in neutrophil anti-*C*. *albicans* functions.

In summary, this study showed that recognition of unopsonized *C*. *albicans* by dectin-2 triggers NET formation through a NADPH oxidase-independent pathway. Signaling pathway leading to NETosis involves Syk-Ca^2+^-PKCδ-NE/PAD4. Dectin-2-mediated NET as revealed in the *C*. *albicans* peritonitis model functions to control fungal spread from peritoneal cavity to kidney. Our work provides a better understanding of the molecular mechanism involved in NADPH oxidase-independent NET formation and sheds light on the role of dectin-2 in neutrophil anti-*C*. *albicans* function.

## Materials and methods

### Fungus and infection

*C*. *albicans* strain SC5314 (ATCC MYA-2876), its isogenic mutant strain HLC54 (yeast-locked strain, *efg1/efg1 cph1/cph1*), GFP-expressing strain OG1 [33], and dTomato-expressing strain CFA2-dTomato [34] were used in this study. All strains were cultured on yeast-peptone-dextrose (YPD) agar (DIFCO) plate at 30°C. Mice were injected intraperitoneally with *C*. *albicans* yeasts prepared in HBSS buffer. Unopsonized *C*. *albicans* was prepared in phenol red free HBSS buffer for experiments. To opsonize, *C*. *albicans* yeasts were added to phenol red free HBSS containing 10% fresh mouse serum and let stand at room temperature for 30 min. To induce hyphal formation, *C*. *albicans* yeasts were incubated in RPMI 1640 medium at 37°C for 4 h before use.

### Mice

Wild-type (C57BL/6), *Itgam*^-/-^, *Ncf-1*^-/-^ (originally purchased from the Jackson Laboratories, Bar Harbor, ME, USA), *Clec7a*^*-/-*^ (from Dr. Gordon Brown, University of Cape Town, Cape Town, South Africa) [35], *Clec4n*^*-/-*^ [19] and MyD88^-/-^ (from Dr. Tsung-Hsien Chuang, National Health Research Institutes, Taiwan) mice were bred and maintained at the Laboratory Animal Center of National Taiwan University College of Medicine. All mice used in this study were maintained under specific pathogen-free conditions. Mice at 6–12 weeks of age were used in all of the experiments.

### Bone marrow neutrophils

Bone marrow cells were harvested from the femurs and suspended in dPBS buffer before overlaid on discontinuous percoll gradients (55%, 62%, and 81% in the order from top to bottom) (GE healthcare). After centrifugation at 1,400 × g for 30 min, cells at the interface between 62% and 81% gradients were harvested and washed. Flow cytometric analysis showed that 90–95% of cells were CD11b^+^Ly6G^+^.

### NET induction and quantification

Two hundred thousand neutrophils suspended in HBSS were seeded in 96 well-plate before addition of 4 × 10^5^ opsonized or unopsonized *C*. *albicans*. The plate was centrifuged at 800 × g for 3 min to spin down cells. Wells were treated with 0.5 U of micrococcal nuclease (MNase, NEB) 3 h later and incubated at 37°C for 10 min to partially digest NET. Supernatants were collected and cell free dsDNA were quantified by Quant-iT PicoGreen dsDNA assay kit (Life technology) according to manufacturer’s instruction.

### Treatment with inhibitor

Neutrophils were pre-treated with indicated inhibitors 30 min before addition of *C*. *albicans*. Inhibitors SkyI (for Syk), BAPTA-AM (a selective chelator of Ca^2+^), Ro 318220 (for total PKC), Rottlerin (for PKCδ), BB-CI-Amidine (for PAD1-4), GSK484 (for PAD4) were all purchased from Cayman. Ro 6976 (for PKCα+β1), LY 333531 (for PKCβ) were from Millipore.

### Flow cytometric analysis of Ca^2+^ influx in neutrophils

One million neutrophils were suspended in 200 μl of phenol red-free HBSS buffer. Loading dye for intracellular Ca^2+^ staining was prepared by adding Calcium Indicator to Signal Enhancer at the ratio of 1:1000 according to the manufacturer’s recommendation (BD Biosciences Calcium Assay Kit, 640176). Two hundred μl of loading dye was added to cells and the mixture was incubated at 37°C for 45 min. After resting in room temperature for 20 min, cells were placed in a 5 ml FACS tube and analyzed by flow cytometry to set the basal level of intracellular Ca^2+^ intensity (30 sec). Unopsonized pre-germinated *C*. *albicans* prepared in 10 μl of HBSS was then added to the tube for continuous flow cytometric analysis for additional 300–420 sec. Data were analyzed by the kinetic mode of FlowJo software. All procedures including sample acquisition and data analysis followed that of modified Bio-protocol published by S. Lee [36]. Original FACS contour plot for measurement of Ca^2+^ intensity is shown in S1 Fig.

### Immunofluorescence staining

Five hundred thousand neutrophils mixed with *C*. *albicans* at a ratio of 1:2 were plated on coverslips and incubated at 37°C for 3 h. Coverslips were fixed in 10% formaldehyde for 15 min and permeabilized with 0.5% Triton X-100. After thorough wash, coverslips were blocked (10% FBS in PBS) and stained with anti-neutrophil elastase (abcam, 1:50) or anti-histone H3 antibody (Cell Signaling, 1:100) at 4°C overnight. Coverslips were stained with cell-permeable DNA dye Hoechst 33258 (1 μg/ml, Invitrogen) or cell-impermeable DNA dye SYTOX Orange (1 μM, Life technology) diluted in blocking buffer and left on ice for 15 min. Coverslips were then mounted by mounting gel and subject to fluorescence microscopic or confocal microscopic analysis.

### Live cell imaging

Five hundred thousand neutrophils suspended in HBSS containing SYTOX Orange (0.5 μM) and cell-permeable DNA dye Draq5 (2 μM) were seeded in a chamber (1 μ-Slide 8 well ibiTreat plates, ibidi). After spun down, 2 × 10^6^ of pre-germinated *C*. *albicans* OG1 were added. NET release was monitored by inverted confocal microscope LSM 780 AxioObserver Z1 for three-color.

### NET fungicidal activity assay

Twenty thousand neutrophils suspended in HBSS were seeded in 96-well plate and allowed to adhere for 30 min before addition of 4 x 10^5^ unopsonized *C*. *albicans* yeasts. Wells containing *C*. *albicans* yeasts without neutrophils were used as control. Plate was centrifuged at 800 × g for 3 min to spin down yeasts. HBSS containing micrococcal nuclease or heat-inactivated MNase (h.i. MNase) was added to the final concentration of 10 U/ml. Buffer was collected and cold H_2_O (pH = 11) was added 3 h later to lyse cells. *C*. *albicans* was detached by mini cell scraper and vigorously pipetting. The number of viable fungi was determined by plating the supernatant on yeast-peptone-dextrose agar plate. Colony counts (CFU) were enumerated 2 days later. The ability of neutrophils to kill *C*. *albicans* is presented as % killing of *C*. *albicans* which was calculated by dividing the difference of CFU counts between the control group (without neutrophils) and neutrophil-added groups by the counts of the control.

### Western blot analysis

Neutrophils stimulated with or without *C*. *albicans* hyphae (MOI = 2) were lysed in PhosphoSafe Extraction Reagent (EMD Millipore). Cell lysates were separated by electrophoresis at 10 or 12.5% SDS-polyacrylamide gel and transferred to Immobilon-P membrane (Millipore). Membrane was blocked with 5% nonfat milk (Fluka) for 1 h and incubated in buffer containing rabbit anti-pSyk (Tyr575), -citrullinated histone H3 (R2 + R8 + R17) (Abcam), -pPKCδ (Ser645) (Cell Signaling), and -β-actin, -GAPDH (GeneTex) antibodies at 4 °C overnight. Membrane was then incubated with buffer containing goat anti-rabbit/rat IgG-HRP antibody (GeneTex) for 1 h. Western Chemiluminescent HRP (Millipore) was used as substrate.

### Peritoneal *C*. *albicans* infection in casein-peritonitis mice

Mice were injected with 1 ml of 9% casein (9 g of casein sodium salt dissolved in 100 ml of hot PBS) intraperitoneally twice with an interval of 18 h. Four hours after the second casein injection, mice were given 1×10^8^ of *C*. *albicans* intraperitoneally. For some experiments, HBSS containing 100 U of micrococcal nuclease or heat-inactivated MNase (h.i. MNase) was administered intraperitoneally at the time of infection. At indicated time after infection, peritoneal exudates, mesentery tissues and kidneys were collected and subject to following experiments.

### In vivo imaging and quantification of fluorescence

Before imaging, mice were injected with 1 ml of 9% casein intraperitoneally twice with an interval of 18 h. Four hours later, mice were infected by 5 × 10^8^ of *C*. *albicans* (SC 5314 or CAF2-dTomato) in 200 μl HBSS intraperitoneally. To monitor NET release, mice were given an additional intraperitoneal injection of 100 μl SYTOX Orange (5 μM) at the time of infection. A Xenogen IVIS Imaging System 200 series (PerkinElemer Inc.) was used to quantify fluorescent *C*. *albicans* (CAF2-dTomato) or the release of extracellular DNA over time. Photons in user-specified region of interest (ROI, gated area) during 10 sec exposure were measured by Living Image 3.2 software. Relative intensity of total photons in ROI was calculated based on the intensity at 1 h after infection.

### Tissue and peritoneal exudate cell immunofluorescence staining

Mesentery were embedded in O.C.T. and allowed to freeze at -80°C overnight. Five μm thick tissue sections were cut and mounted on gelatin-coated slides. Peritoneal exudates were diluted 1:5 and seeded on coverslips for 1 h. Slides and coverslips were fixed with 4% paraformaldehyde, left on ice for 30 min, permeabilized with 0.5% Triton X-100 and let sit at room temperature for 5 min. After wash, samples were blocked (PBS containing 5% FBS) and stained with anti-histone H3 (Cell Signaling), -Ki67 and -Ly6G (Biolegend) antibodies at 4°C overnight. Nucleus was stained with Hoechst 33258 (1 μg/ml, Invitrogen) for 15 min. Samples were then mounted by mounting gel and subject to confocal microscopic analysis.

### Quantification of fungal load

Kidneys were collected and homogenized in a tissue grinder with 1 ml of RPMI 1640 medium. Peritoneal exudate was collected from mice after intraperitoneal injection of 5 ml of HBSS. Homogenates and peritoneal fluids were treated with 0.5 U of micrococcal nuclease and incubated at 37°C for 10 min to digest DNA entangled with fungus. One hundred microliter of kidney homogenates and peritoneal exudates were plated on YPD agar. Colonies were counted after incubation at 30°C for 2–3 days.

### Ki67 staining

Peritoneal exudates from naïve and infected mice were collected, treated with 0.5 U/ml MNase and subject to surface staining for neutrophil marker CD11b and Ly6G. After fixation in 4% paraformaldehyde and permeabilization with 1% saponin, cells were stained with anti-Ki67 antibody prepared in staining buffer (0.5% saponin) overnight. Cells were fixed in 1% PFA and subject to flow cytometric analysis.

### Statistics

Student *t* test was used to compare the difference between two groups. Statistical significance was defined as *P* < 0.05.

### Ethics statement

Mouse study was carried out in strict accordance with the recommendations in the Guidebook for the Care and Use of Laboratory Animals, The Third Edition, 2007, published by The Chinese-Taipei Society of Laboratory Animal Sciences. All animal procedures and experimental protocols were approved by AAALAC-accredited facility, the Committee on the Ethics of Animal Experiments of the National Taiwan University College of Medicine (Permit Number: 20140304, 20140533 and 20180013).

## Supporting information

S1 Fig The contour plot of calcium influx in neutrophils upon unopsonized *C*. *albicans* stimulation and the gating strategy for % Ca^2+^-positive cells. Cells were loaded with Ca^2+^ indicator and incubated for 45 min. After resting, cells were analyzed by flow cytometry to set the basal level of intracellular Ca^2+^ intensity (30 sec). Cell was then stimulated or not (Ctrl.) with unopsonized pre-germinated *C*. *albicans* (*Ca* MOI = 4) (Max response, set as 100%) and subject to continuous flow cytometric analysis for additional 300 sec. Contour plot on the left shows the intensity of intracellular Ca^2+^ intensity over the time course of the experiment. Histogram on the right of the contour plot shows % Ca^2+^-positive cells at 20–50 sec.
(TIF)

S2 Fig Neutrophil elastase (NE) translocation in *Ncf-1*^*+/+*^ and *Ncf-1*^*-/-*^ neutrophils upon stimulation by unopsonized *C*. *albicans*. *Ncf-1*^*+/+*^ and *Ncf-1*^*-/-*^ neutrophils were seeded on coverslips and stimulated with unopsonized *C*. *albicans* at MOI of 2. At indicated times after stimulation, cells were permeabilized and stained with anti-neutrophil elastase antibody (green) and cell-permeable DNA dye Hoechst 33258 (blue). Immunofluorescence images were viewed under fluorescence microscope.
(TIF)

S3 Fig NETotic response of *Ncf-1*^*+/+*^ and *Ncf-1*^*-/-*^ mice to peritoneal *C*. *albicans* infection. *Ncf-1*^*+/+*^ and *Ncf-1*^*-/-*^ mice were injected with two doses of 9% casein intraperitoneally. At 4 h after second injection, mice were given *C*. *albicans* (1 × 10^8^) intraperitoneally. At 3 h after infection, peritoneal exudates, mesenteric tissues and kidneys were collected. (A) Peritoneal exudates were seeded on coverslips and incubated for 1 h. Cells were then permeabilized and stained for Ki67 (orange), histone H3 (red), Ly6G (green) and nucleus (blue) and viewed under fluorescence microscope. DIC, differential interference contrast image. Arrows point to Ki67^+^ cells. (B) Mesenteric tissues were collected and embedded in O.C.T. Cryosections were stained for Ki67 (red), Ly6G (green) and nucleus (blue) and viewed under fluorescence microscope. (C) Fungal counts in total peritoneal fluid and kidney homogenates were determined by plating. Fungal colonies were counted 2–3 days later. ***, *p <* 0.005, as analyzed by Student’s *t* test.
(TIF)

S4 Fig NETotic response of *clec4n*^*+/+*^ and *clec4n*^*-/-*^ mice to peritoneal *C*. *albicans* infection. (A) Peritoneal exudates were harvested from *clec4n*^*+/+*^ and *clec4n*^*-/-*^ mice at 4 h after receiving two peritoneal injections of 9% casein. Total numbers of peritoneal cells from *clec4n*^*+/+*^ and *clec4n*^*-/-*^ mice are shown on the left. Cells were stained with anti-Ly6G, -CD11b, and -Ki67 antibodies and subject to flow cytometric analysis. % of Ly6G^+^ cells (neutrophils) in total peritoneal cell population are shown on the right. (B) Peritoneal exudates were harvested from *clec4n*^*+/+*^ and *clec4n*^*-/-*^ mice with (*Ca*) or without (naïve) *C*. *albicans* infection. Cells were stained as described in (A). Gating strategy for CD11b, Ly6G and Ki67 is shown in dot pot. Histograms show Ki67 intensity in the CD11b^+^Ly6G^+^ neutrophil population.
(TIF)

S1 Video Stimulation of *Ncf-1*^*+/+*^ neutrophils by opsonized *C*. *albicans* triggers NET formation. *Ncf-1*^*+/+*^ neutrophils were stained with cell-permeable DNA dye Draq5 (blue) and cell-impermeable DNA dye SYTOX Orange (red) before stimulation with opsonized pre-germinated GFP-expressing *C*. *albicans* strain OG1 (green). NETosis in response to opsonized pre-germinated *C*. *albicans* was observed over 180 min after addition of *C*. *albicans*.
(MOV)

S2 Video Stimulation of *Ncf-1*^*+/+*^ neutrophils by unopsonized *C*. *albicans* triggers NET formation. *Ncf-1*^*+/+*^ neutrophils were stained with cell-permeable DNA dye Draq5 (blue) and cell-impermeable DNA dye SYTOX Orange (red) before stimulation with unopsonized pre-germinated GFP-expressing *C*. *albicans* strain OG1 (green). NETosis in response to unopsonized pre-germinated *C*. *albicans* was observed over 180 min after addition of *C*. *albicans*.
(MOV)

S3 Video Stimulation of *Ncf-1*^*-/-*^ neutrophils by unopsonized *C*. *albicans* triggers NET formation. *Ncf-1*^*-/-*^ neutrophils were stained with cell-permeable DNA dye Draq5 (blue) and cell-impermeable DNA dye SYTOX Orange (red) before stimulation with unopsonized pre-germinated GFP-expressing *C*. *albicans* strain OG1 (green). NETosis in response to unopsonized pre-germinated *C*. *albicans* was observed over 180 min after addition of *C*. *albicans*.
(MOV)
