# Supplementary figures and images for: Candida albicans triggers NADPH oxidase-independent neutrophil extracellular traps through dectin-2
Source: PLoS Pathog. 2019 Nov 6;15(11):e1008096. doi: 10.1371/journal.ppat.1008096 (PMC6834254; doi:10.1371/journal.ppat.1008096)

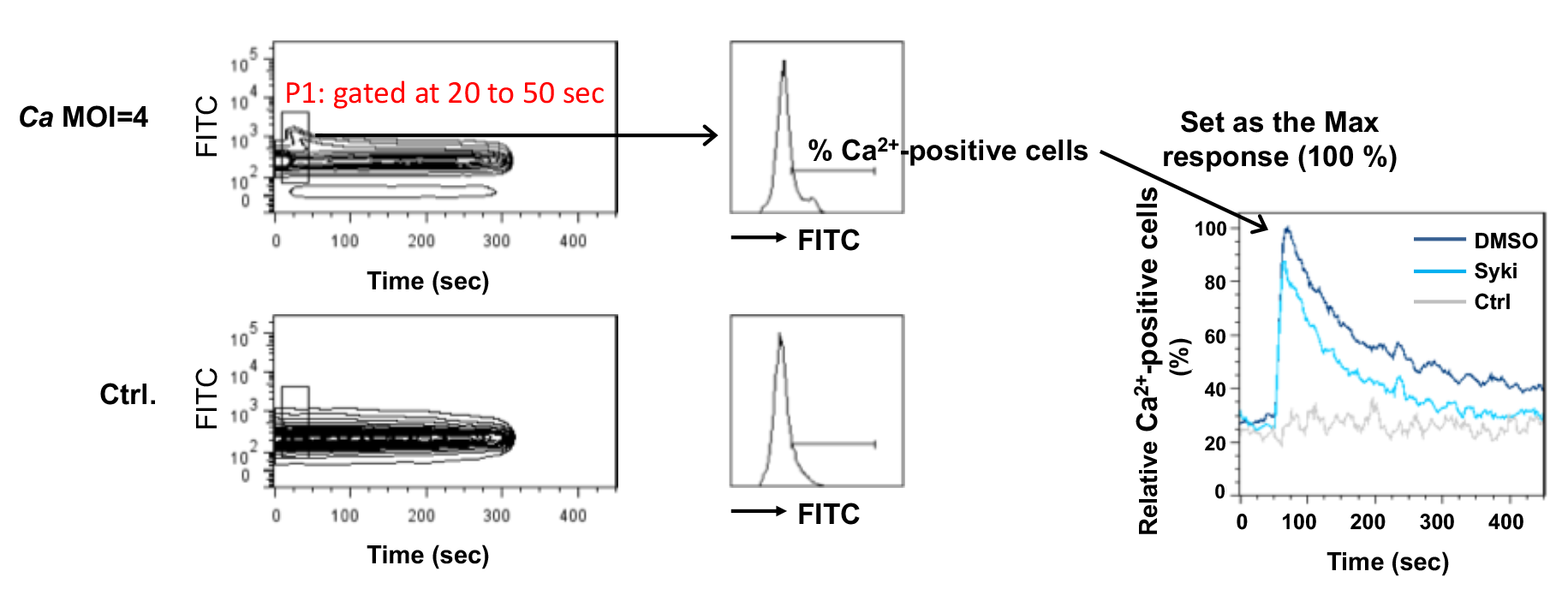

Supplement: S1 Fig — Cells were loaded with Ca2+ indicator and incubated for 45 min. After resting, cells were analyzed by flow cytometry to set the basal level of intracellular Ca2+ intensity (30 sec). Cell was then stimulated or not (Ctrl.) with unopsonized pre-germinated C. albicans (Ca MOI = 4) (Max response, set as 100%) and subject to continuous flow cytometric analysis for additional 300 sec. Contour plot on the left shows the intensity of intracellular Ca2+ intensity over the time course of the experiment. Histogram on the right of the contour plot shows % Ca2+-positive cells at 20–50 sec. (TIF) [file ppat.1008096.s001.tif]

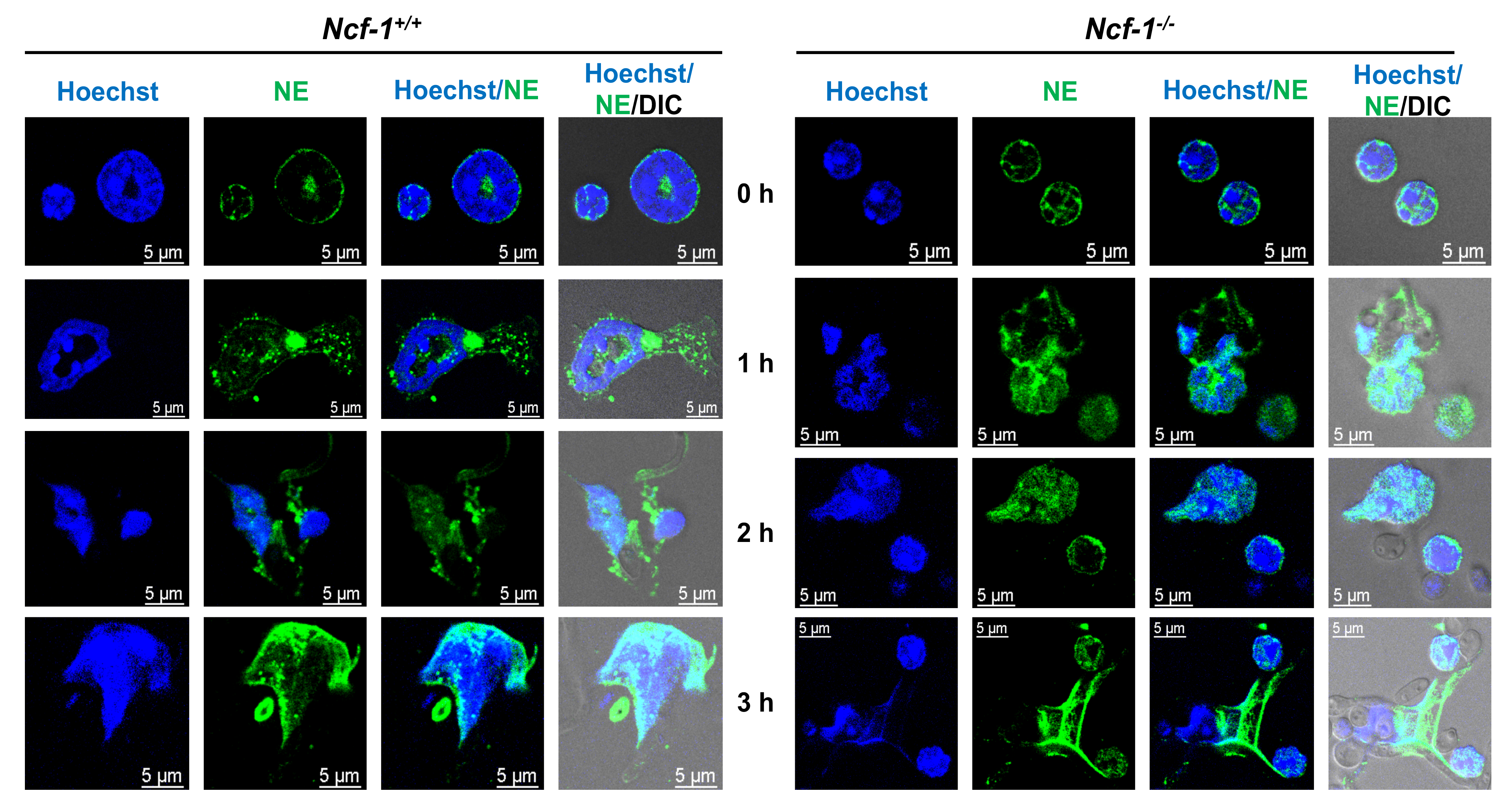

Supplement: S2 Fig — Ncf-1+/+ and Ncf-1-/- neutrophils were seeded on coverslips and stimulated with unopsonized C. albicans at MOI of 2. At indicated times after stimulation, cells were permeabilized and stained with anti-neutrophil elastase antibody (green) and cell-permeable DNA dye Hoechst 33258 (blue). Immunofluorescence images were viewed under fluorescence microscope. (TIF) [file ppat.1008096.s002.tif]

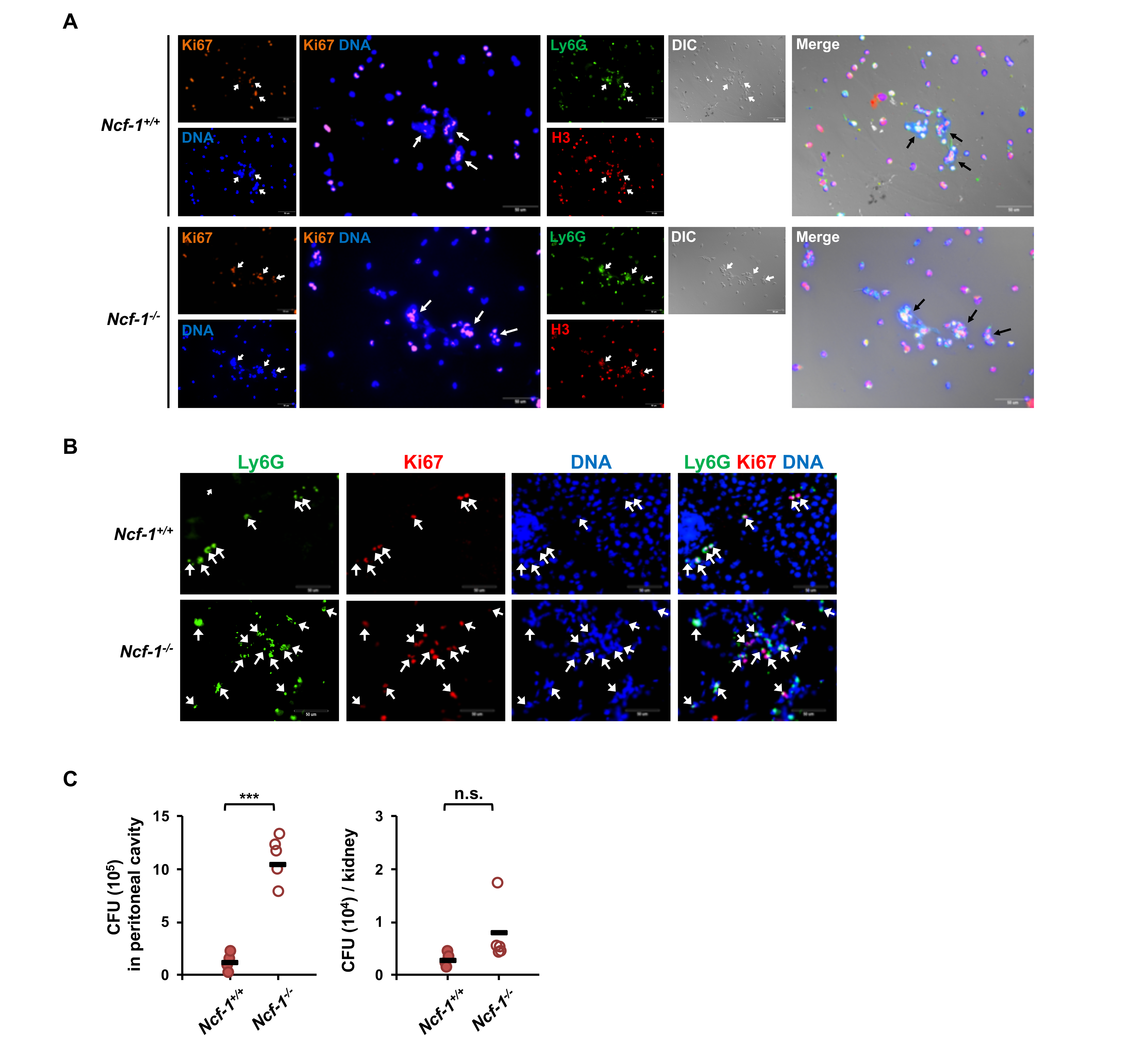

Supplement: S3 Fig — Ncf-1+/+ and Ncf-1-/- mice were injected with two doses of 9% casein intraperitoneally. At 4 h after second injection, mice were given C. albicans (1 × 10^8^) intraperitoneally. At 3 h after infection, peritoneal exudates, mesenteric tissues and kidneys were collected. (A) Peritoneal exudates were seeded on coverslips and incubated for 1 h. Cells were then permeabilized and stained for Ki67 (orange), histone H3 (red), Ly6G (green) and nucleus (blue) and viewed under fluorescence microscope. DIC, differential interference contrast image. Arrows point to Ki67+ cells. (B) Mesenteric tissues were collected and embedded in O.C.T. Cryosections were stained for Ki67 (red), Ly6G (green) and nucleus (blue) and viewed under fluorescence microscope. (C) Fungal counts in total peritoneal fluid and kidney homogenates were determined by plating. Fungal colonies were counted 2–3 days later. ***, p < 0.005, as analyzed by Student’s t test. (TIF) [file ppat.1008096.s003.tif]

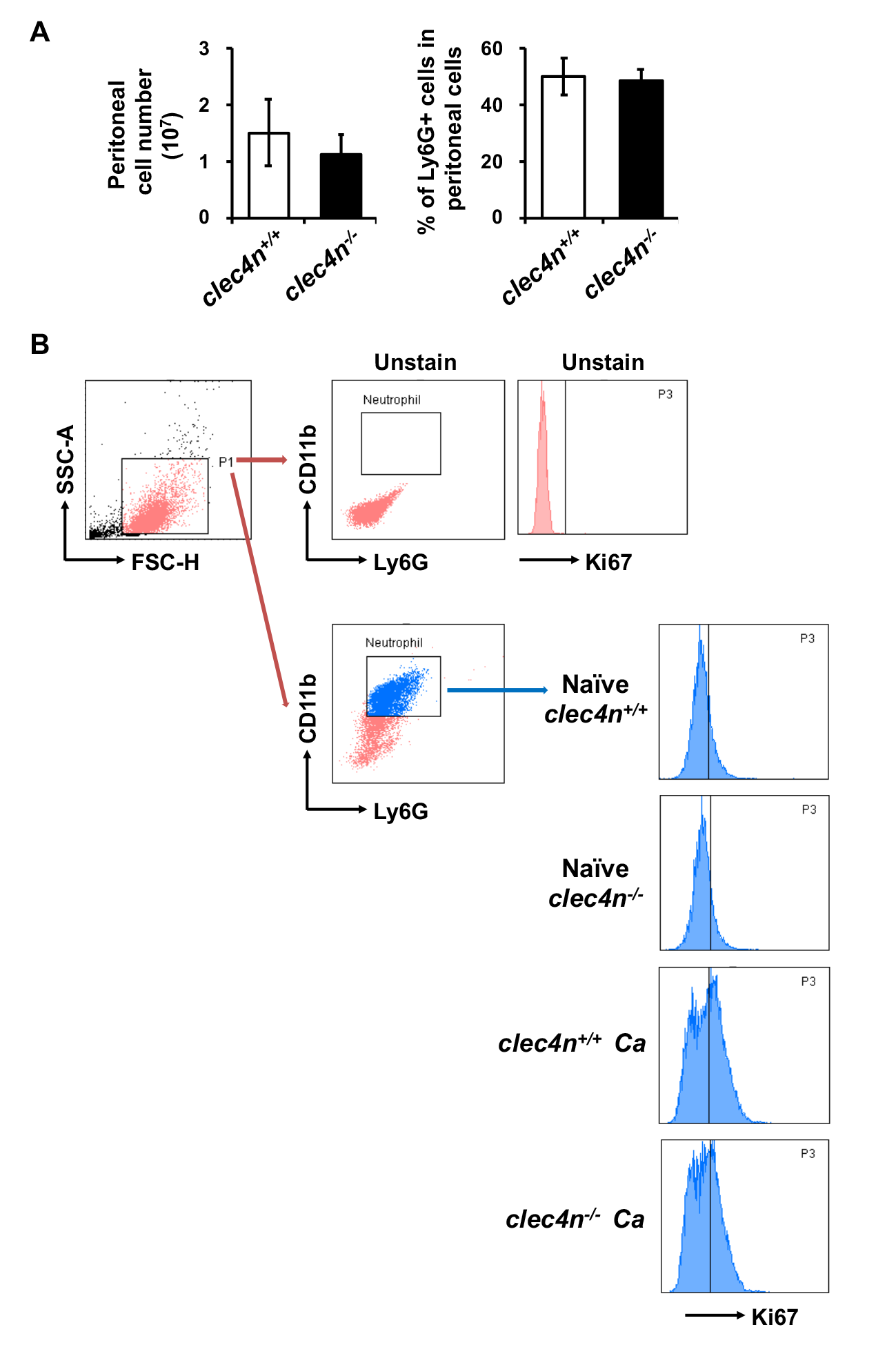

Supplement: S4 Fig — (A) Peritoneal exudates were harvested from clec4n+/+ and clec4n-/- mice at 4 h after receiving two peritoneal injections of 9% casein. Total numbers of peritoneal cells from clec4n+/+ and clec4n-/- mice are shown on the left. Cells were stained with anti-Ly6G, -CD11b, and -Ki67 antibodies and subject to flow cytometric analysis. % of Ly6G+ cells (neutrophils) in total peritoneal cell population are shown on the right. (B) Peritoneal exudates were harvested from clec4n+/+ and clec4n-/- mice with (Ca) or without (naïve) C. albicans infection. Cells were stained as described in (A). Gating strategy for CD11b, Ly6G and Ki67 is shown in dot pot. Histograms show Ki67 intensity in the CD11b+Ly6G+ neutrophil population. (TIF) [file ppat.1008096.s004.tif]
